# Development of a low-cost and high-throughput LC–MS method for newborn screening of thalassemia and abnormal hemoglobin disorders

**DOI:** 10.1007/s12519-025-00962-y

**Published:** 2025-09-01

**Authors:** Wen-Xia Huang, Yu-Xin Cai, Jing Yang, Su-Rong Fu, Ming Wang, Juan Zhang, Ke-Xing Wan, Chao-Wen Yu

**Affiliations:** 1 Center for Clinical Molecular Medicine & Newborn Screening, Children’s Hospital of Chongqing Medical University; National Clinical Research Center for Child Health and Disorders; Ministry of Education Key Laboratory of Child Development and Disorders, No.136 Zhongshan 2nd Road, Yuzhong District, Chongqing, 400014 China; 2 Chongqing Engineering Research Center of Stem Cell Therapy, Chongqing, 400014 China; 3 Chongqing Key Laboratory of Child Rare Diseases in Infection and Immunity, Chongqing, 400014 China; 4 Chongqing University Fuling Hospital, Chongqing, 408000 China

**Keywords:** Hemoglobin (Hb), Hemoglobinopathy, High resolution mass spectrometry (HRMS), Thalassemia, Newborn screening

## Abstract

**Background:**

Screening and pre-symptomatic diagnosis in newborns allows early treatment of thalassemia and abnormal hemoglobin (Hb) disorders in childhood. However, there remains a lack of efficient methods to screen for hemoglobinopathies in newborns. This study aimed to establish a bottom-up mass spectrometry (MS)-based method for efficient screening of hemoglobinopathies in newborns using dried blood spot (DBS) samples.

**Methods:**

We developed LC–MS methodology using high-performance liquid chromatography (HPLC) combined with high-resolution mass spectrometry (HRMS). DBS samples from patients covering the most common types of hemoglobinopathies and normal controls were collected. We extracted Hb from a 3.2 mm disc punched from the DBS sample, which was then digested with trypsin to release a series of Hb-specific peptides. Using HPLC–HRMS, we identified disease-related peptides for biomarker design. Using this methodology, we built a prediction model using binary logistic regression to facilitate efficient screening.

**Results:**

This new method costs less than $1 per test and can process at least 192 samples per batch. Our methodology is fast with a sampling and analysis time of 2.6 minutes and inter- and intra-assay coefficients of variation below 14.67%. Moreover, we report low limits of quantification for the proteo-specific peptides (0.50–60.00 μg/L). No significant matrix effects or carryover were observed. Our method could give reliable results even with DBS samples stored for one month. Prospective application of this method to 2726 newborns identified 87 patients with hemoglobinopathies and achieved high screening sensitivity and specificity for deletional α-thalassemia (--^SEA^) (100.00% and 100.00%), *β*-thalassemia (97.50% and 89.63%) and other abnormal Hb disorders.

**Conclusions:**

We have developed a low-cost, high-throughput method for reliable screening of thalassemia and abnormal Hb disorders in newborns. This could be deployed as a first-line screening test.

**Graphical abstract:**

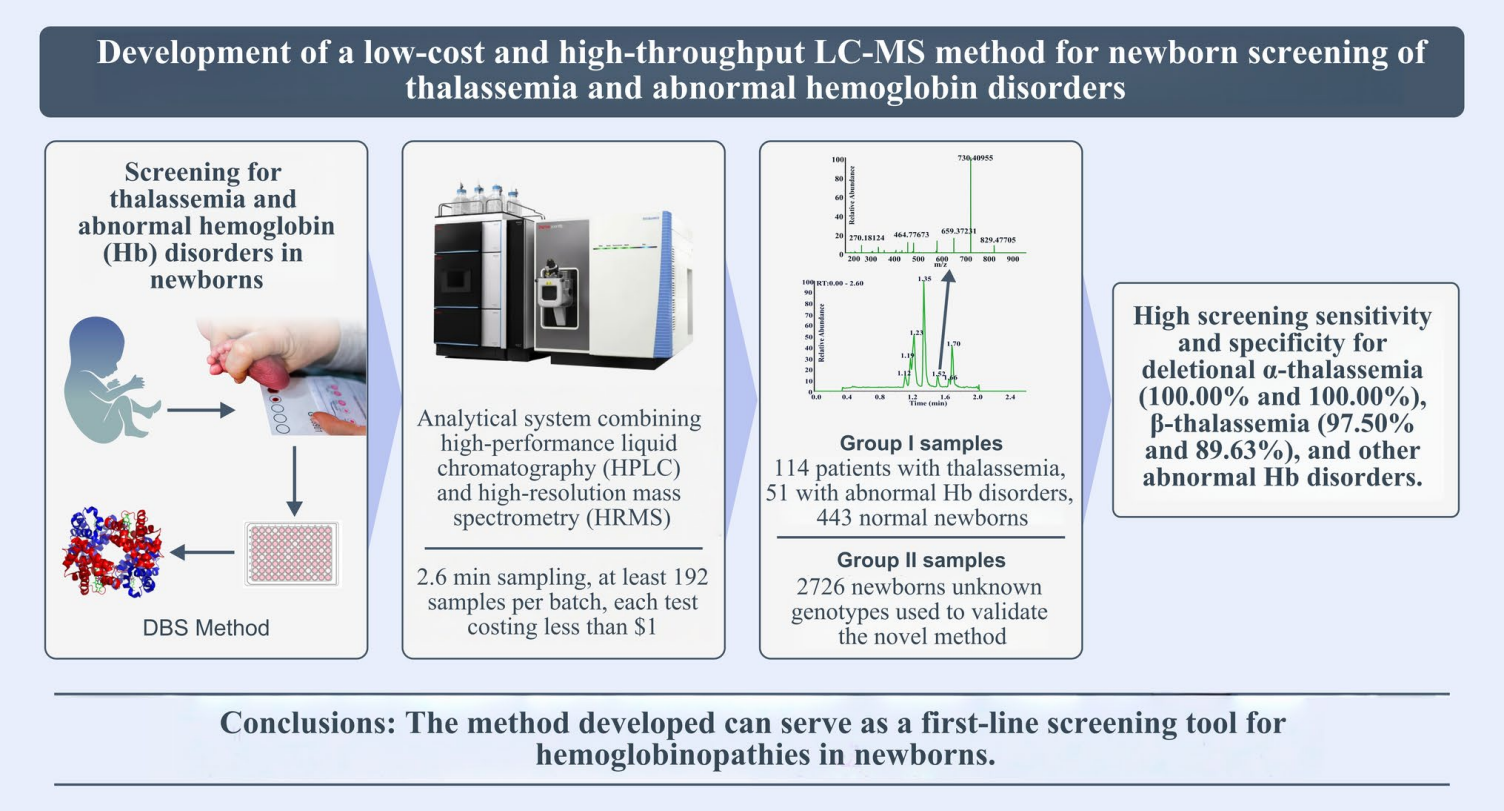

**Supplementary Information:**

The online version contains supplementary material available at 10.1007/s12519-025-00962-y.

## Introduction

Hemoglobinopathies, including α-thalassemia, β-thalassemia, abnormal hemoglobin (Hb) disorders and the hereditary persistence of fetal hemoglobin (HPFH), are the most common monogenic diseases. These are caused by the abnormal synthesis of Hb chains in red blood cells (RBCs) and resulting in a variety of clinical problems [1]. Newborn screening provides an opportunity for pre-symptomatic diagnosis and early treatment of hemoglobinopathies, preventing babies from developing severe symptoms [2–5]. Existing hemoglobinopathy screening tests, including high-performance liquid chromatography (HPLC), isoelectric focusing electrophoresis (IEF) and capillary zone electrophoresis (CZE) [6, 7] are based on the structural integrity of Hb, which are easily affected by sample hemolysis or Hb degradation [8]. In addition, post-translational modification can also affect the elution or migration rates of Hb further complicating data interpretation [9–11].

In the past decade, researchers have attempted to establish a mass spectrometry (MS)-based method to screen for hemoglobinopathies in newborns [12–14]. However, most attempts did not achieve efficient and simultaneous screening of α-thalassemia, β-thalassemia and abnormal Hb disorders using dried blood spot (DBS) samples. This is probably because α-like and β-like globin chain expression changes quickly after birth. In addition, the rare components of Hb are difficult to be quantified in only one piece of a DBS sample. In the present study, we analyzed the globin chains expressed in the first few days post-partum, identified the most informative proteo-specific peptides for biomarker design and established a bottom-up MS-based method for efficient screening of hemoglobinopathies in newborns using DBS samples.

## Methods

### Samples and chemicals

The Institutional Ethical Review Boards of the Children’s Hospital of the Chongqing Medical University approved this study (No. 2018-64). Two cohots were included for the analysis. Group I (development cohort, *n* = 608) consisted of 114 patients with thalassemia, 51 with abnormal Hb disorders, and 443 normal newborns. All samples from group I underwent genotyping via next-generation sequencing (NGS), they were then used for biomarker selection, screening method establishment and definition of reference intervals (Table 1). Group II (validation cohort, *n* = 2726) consisted of newborns with unknown genotypes and was used to validate the newly developed screening method. All DBS samples were prepared from either peripheral blood applied to filter cards or direct heel stick collections and were stored at 4 °C until analysis.

**Table 1:** The screening cut-offs generated for thalassemia and abnormal Hb disorders

Genotypes^*a*^	Number	Age	Screening cutoffs^*b*^					Sensitivity^*c*^ (%)	Specificity^*c*^ (%)
			*P*(α)	*P*(β)		*G*_M_/β_T1_			
Normal individuals	443	2–30 d	< 0.33		< 0.13		0.00	–	–
α-thalassemia	79	3–30 d	≥ 0.33		–		–	86.08	94.13
α^+^	41	3–30 d	≥ 0.33		–		–	80.49	94.13
αα/-α^3.7^; β^N^/β^N^	26	3–30 d	≥ 0.35		–		–	69.23	94.36
αα/-α^4.2^; β^N^/β^N^	15	17–30 d	≥ 0.33		–		–	100.00	94.13
α^0^	17	4–30 d	≥ 0.66		–		–	100.00	99.55
αα/--^SEA^; β^N^/β^N^	16	4–30 d	≥ 0.87		–		–	100.00	100.00
-α^3.7^/-α^3.7^; β^N^/β^N^	1	30 d	≥ 0.66		–		–	100.00	99.55
Non-deletional	16	3–30 d	≥ 0.40		–		–	81.25	95.03
αα/α^CS^α; β^N^/β^N^	9	17–30 d	≥ 0.49		–		–	88.89	97.29
αα/α^QS^α; β^N^/β^N^	1	28 d	≥ 0.64		–		–	100.00	99.55
αα/α^WS^α; β^N^/β^N^	5	30 d	≥ 0.40		–		–	80.00	95.03
αα/α^Groene Hart^α; β^N^/β^N^	1	3 d	≥ 0.40		–		–	0.00	95.03
HbH	5	11–30 d	≥ 0.88		–		–	100.00	100.00
--^SEA^/-α^3.7^; β^N^/β^N^	2	29–30 d	≥ 0.88		–		–	100.00	100.00
--^SEA^/-α^4.2^; β^N^/β^N^	3	11–30 d	≥ 0.88		–		–	100.00	100.00
β-thalassemia	32	2–30 d	–		≥ 0.13		–	90.63	96.84
β^+^	10	3–30 d	–		≥ 0.13		–	80.00	96.80
αα/αα; β^N^/β^IVS-II-654^	8	6–30 d	–		≥ 0.13		–	100.00	96.80
αα/αα; β^N^/β^3'UTR +129 (T>A)^	2	3–23 d	–		≥ 0.13		–	0.00	96.80
β^0^	22	2–30 d	–		≥ 0.20		–	95.40	97.70
αα/αα; β^N^/β^Codon 17^	3	6–29 d	–		≥ 0.22		–	100.00	97.90
αα/αα; β^N^/β^Codons 41/42^	16	3–30 d	–		≥ 0.20		–	93.70	97.70
αα/αα; β^N^/β^Codons 71/72^	2	2–30 d	–		≥ 0.29		–	100.00	98.40
αα/αα; β^N^/β^Codon 43^	1	30 d	–		≥ 0.86		–	100.00	100.00
α- and β-thalassemia	3	16–30 d	≥ 0.33		≥ 0.13		–	100.00	96.80
αα/-α^3.7^; β^N^/β^-28^	1	30 d	≥ 0.46		≥ 0.85		–	100.00	100.00
αα/α^WS^α; β^N^/β^IVS-II-654^	1	16 d	≥ 0.33		≥ 0.88		–	100.00	100.00
αα/--^SEA^; β^N^/β^-28^	1	30 d	≥ 0.56		≥ 0.88		–	100.00	100.00
Abnormal Hb disorders	51	4 d-17 y	–		–		> 0.00	100.00	100.00
Hb S	3	3 m-5 y	–		–		> 0.00	100.00	100.00
Hb E	40	25 d-17 y	–		–		> 0.00	100.00	100.00
Hb D-Los Angeles	4	7 d-10 y	–		–		> 0.00	100.00	100.00
Hb G-Coushatta	1	6 d	–		–		≥ 2.19	100.00	100.00
Hb Watford	1	4 d	–		–		> 0.00	100.00	100.00
Hb Nanchang	1	4 d	–		–		> 0.00	100.00	100.00
Hb Hekinan II	1	7 d	–		–		> 0.00	100.00	100.00

^*a*^HGVS nomenclature: -α^3.7^ = NC_000016.9:g.223300_227103del. -α^4.2^ = NC_000016.9:g.219817_(223755_ 224,074)del. --^SEA^ = NC_000016.9:g.215400_234700del. α^CS^α = HBA2:c.427 T > C. α^QS^α = HBA2:c.377 T > C. α^WS^α = HBA2:c.369C > G. α^Groene Hart^α = HBA1:c.358C > T. β^IVS-II-654^ = HBB: c.316-197C > T. β^3'UTR +129 (T>A)^ = HBB: c.129 T > A. β^Codon 17^ = HBB: c.52A > T. β^Codons 41/42^ = HBB: c.126_129delCTTT. β^Codons 71/72^ = HBB: c.216_217insA. β^Codon 43^ = HBB: c.130G > T. β^-28^ = HBB: c.-78A > G. Hb S = HBB: c.20A > T. Hb E = HBB: c.79G > A. Hb D-Los Angeles = HBB: c.364G > C. Hb G-Coushatta = HBB: c.68A > C. Hb Watford = HBB: c.5 T > G. Hb Nanchang = HBA2: c.46G > A. Hb Hekinan II = HBA1:c.84G > T

*d* days, *m* months, *y* years, *Hb* hemoglobin, "*–*" no data

^*b*^The screening cutoffs were determined based on the current study groups, that may be different in other laboratories

^*c*^The sensitivity and the specificity of the method were evaluated by comparing the patients with the normal individuals recruited in the current study groups

HPLC-grade acetonitrile (CAS 75-05-8) and methanol (CAS 67-56-1) were obtained from Honeywell, South Korea and stored at room temperature (RT). Formic acid (CAS 64-18-6, Sigma-Aldrich, Darmstadt, Germany) with a purity of 95% was stored at RT. We prepared 5 g/L trypsin (CAS 9002-07-7, Sigma-Aldrich, St. Louis, MO, USA) by dilution with 1 mol/L ammonium bicarbonate solution (CAS 1066-33-7, Sigma-Aldrich, Darmstadt, Germany); this was stored at − 20 °C. The denaturing solution was prepared using formic acid, methanol, acetonitrile and deionized water (1:80:200:520), stored at RT. Internal standards (IS) and stable isotope-labeled peptides (synthesized by GL Biochem Ltd, Shanghai, China, with a purity of 99% using Fmoc-Lys^13C,15N^ –OH) were diluted to a concentration of 1 mg/mL with deionized water containing 25% acetonitrile and stored at − 20 °C. We used the Sebia capillary electrophoresis device (Sebia-CE, Capillarys 2 Neonat Fast™, Evry, France) and a commercial kit (Capillarys Minicap Hemoglobin E) as the reference method for screening hemoglobinopathies.

### Sample preparation

Disks with a diameter of 3.2 mm were punched from the DBS samples and transferred to a 96-well plate, followed by the addition of 200 µL of deionized water containing 20% of methanol, then gentle shaking for 30 minutes at RT. Subsequently, 50 µL of the sample lysates were transferred to a new 96-well plate, supplemented with 40 µL of denaturing solution prior to 5 minutes of incubation. A total of 10 µL of trypsin (5 g/L) was added to each well, sealed with a sealing film and incubated at 37 °C for 1 hour. 15 µL of digested solution was transferred from each well to a 96-well filter plate (MSGVN2250, Sigma-Aldrich, Darmstadt, Germany), diluted with 120 µL of deionized water containing acetonitrile (1:9) and 15 µL of IS (2 mg/L α_T1_ IS, 10 mg/L α_T3_ IS, 5 mg/L β_T1_ IS, 5 mg/L β_T2_ IS, 0.5 mg/L γ_T10_ IS, 0.2 mg/L δ_T2_ IS and 0.1 mg/L ζ_T8_ IS), then centrifuged (600× *g*) at 4 °C for 2 minutes. The filtered solutions were ultimately transferred to a new 96-well plate, ready for analysis.

### High performance liquid chromatography-high resolution mass spectrometry (HPLC–HRMS) assay

To enhance detection sensitivity the diluted samples containing the digested peptides were separated on an Ultimate 3000 system using a Hypersil GOLDTM C18 UHPLC column (100 × 4.6 mm, 1.9 μm, Thermo ScientificTM). Two mobile phases were used: mobile phase A was deionized water containing formic acid (0.1%), while mobile phase B was HPLC-grade acetonitrile containing formic acid (0.1%). To improve peptide separation, we used a gradient flow (Supplemental Table 1). MS analysis was performed using a Quadrupole-Orbitrap MS system (Thermo Q Exactive Focus) with an electrospray ionization source connected to the Ultimate 3000 system (Supplemental Table 2). Each peptide underwent collision-induced dissociation, and the resulting precursor-product ion pairs (mass transitions) were monitored in positive-ion mode using parallel reaction monitoring (PRM) (Supplemental Table 3).

### Identification of proteo-specific peptides for the screening assay

Tryptic digestion of the Hb subunits yielded a series of proteo-specific peptides that were separated and identified by HPLC–HRMS within 2.6 min. The most informative peptides (signal-to-noise ratio ≥ 10) were selected to represent the corresponding globin chains and determine their relative concentrations using stable isotope-labeled peptides as IS (Eq. F1). To design effective screening markers for thalassemia, the ratios of proteo-specific peptides were calculated to reflect the degree of imbalance between α-like and β-like globin chains, or the proportion of rare Hb subunits compared with the main components (Eq. F2). For screening of abnormal Hb disorders, the ratio of the mutant globin chain to the wild-type *β* chain (*G*_M_/*β*) (Eq. F3) was calculated.

F1 $${C}_{\zeta Tn}=\frac{\zeta Tn\, Area * C\zeta Tn\, IS }{ \zeta Tn\, IS\, Area }$$

F2 $$\upzeta /\beta ={C}_{\zeta Tn}/{C}_{\beta Tm}=\frac{\zeta Tn\, Area * \beta Tm\, IS\, Area *C\, \zeta Tn\, IS}{ \zeta Tn\, IS\, Area * \beta Tm\, Area *C\,\beta Tm\, IS}$$

F3 $${G}_{\text{M}}/\beta =\frac{GMTn\,Area * \beta Tn\,IS\,Area }{ GMTn\,IS\,Area * \beta Tn\,Area}$$

where *ζTn Area* is the mass spectra peak area (MSPA) of peptide Tn from the ζ chain; *ζTn IS Area* is the MSPA of the IS peptide Tn; *C*_*ζTn*_* IS* is the standard concentration of the IS peptide Tn; *βTm Area* is the MSPA of peptide Tm from the *β* chain; *βTm IS Area* is MSPA of the IS peptide Tm; *C*_*βTm*_* IS* is the standard concentration of the IS peptide Tm; *G*_M_*Tn Area* is the MSPA of the mutant peptide Tn from the globin chain, *G*_M_*Tn IS Area* is the MSPA of the IS peptide Tn; *βTn Area* is the MSPA of the wild-type peptide Tn from the *β* chain; and *βTn IS Area* is the MSPA of the IS peptide Tn.

### Determination of screening cut-offs for thalassemia and abnormal Hb disorders

All 608 samples from Group I were analyzed using HPLC–HRMS. Globin chain ratios were obtained using the above equations and their potential value for disease discrimination was determined by receiver operating characteristic (ROC) curve analysis using GraphPad Prism 9.5. Peptides with globin ratios with an area under the curve (AUC) value exceeding 0.850 were selected as candidate biomarkers. To enhance the sensitivity and specificity of the HPLC–HRMS approach, we developed a prediction model through binary logistic regression, aiming to refine the screening cut-offs and normal reference intervals. The optimized screening cut-offs were finally determined by maximizing the Youden index (Youden index = sensitivity + specificity − 1).

### Method validation

Our methodology was evaluated using a standard protocol for clinical MS [15]. Lower limits of quantitation (LLOQ) were assessed by measuring native matrix samples prepared with serum only. Inter and intra-assay variability was assessed using quality control samples at different concentrations on three consecutive days. To investigate matrix effects, samples prepared with trypsin-digested serum, normal saline and a mixture of trypsin-digested serum and normal saline (1:1) were compared. Carryover rate was assessed by first testing samples prepared with high concentrations of selected peptides and then testing the samples prepared with low concentrations. The recovery of the selected peptides was assessed at three different concentrations by spiking standard peptides into trypsin-digested serum with known concentrations. Stability of the DBS samples stored under different conditions (RT, 4 °C, and − 20 °C) was also evaluated.

### Evaluation of clinical utility

We screened all 2726 samples from Group II using HPLC–HRMS and Sebia-CE in parallel. The HPLC–HRMS approach used the optimized screening cut-offs, whereas the Sebia-CE method adhered to the recently recommended decision-making rules (Supplemental Table 4) [16]. Using NGS, we validated all cases that screened positive by HPLC–HRMS or Sebia-CE. Subsequently, we evaluated the sensitivity and specificity of the two methods to demonstrate their efficacy in screening for hemoglobinopathies in newborns.

## Results

### Proteo-specific peptides selected for the screening assay

**Fig. 1:**
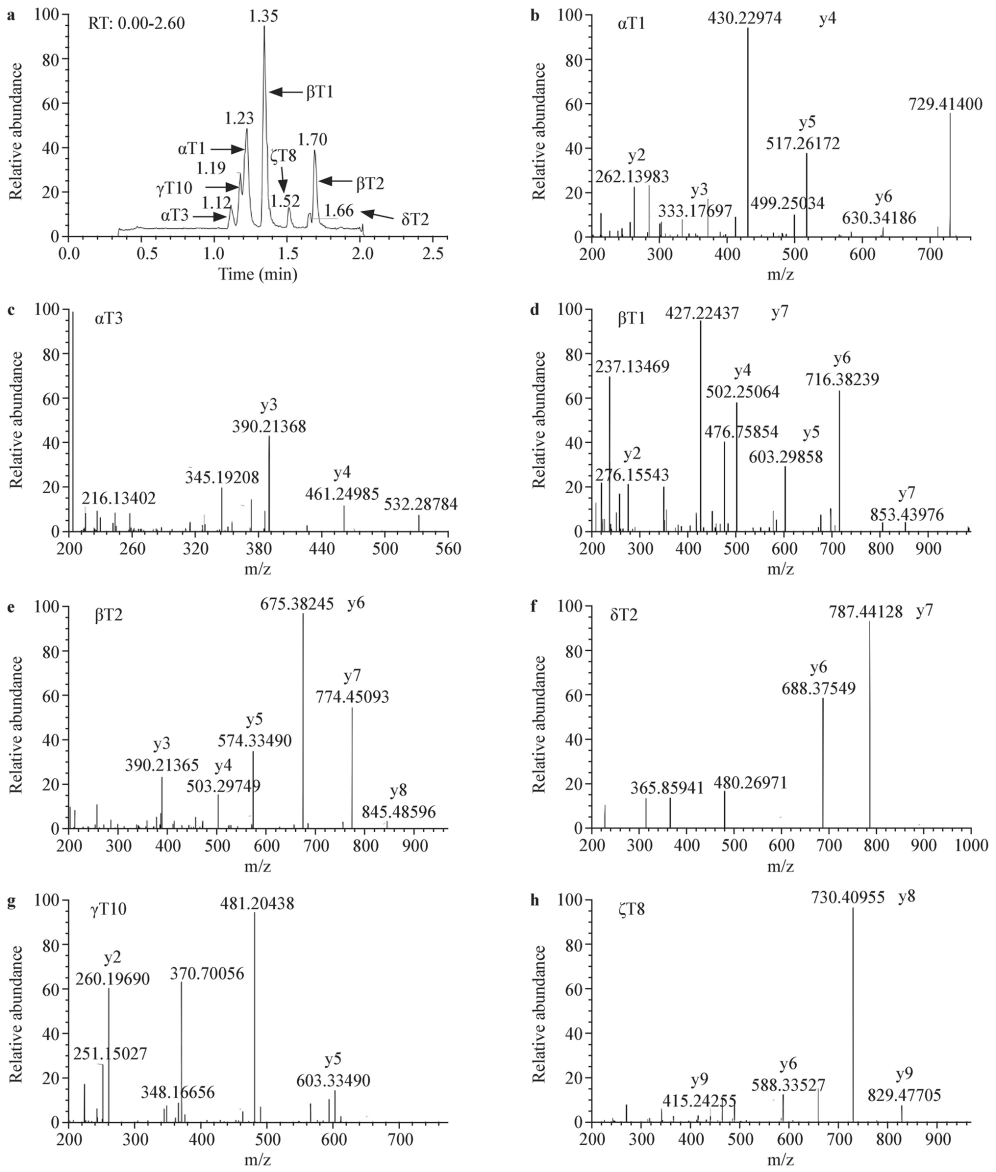
Separation and identification of selected proteo-specific peptides. **a** Pre-separation of the selected proteo-specific peptides (α_T1_, α_T3_, β_T1_, β_T2_, γ_T10_, δ_T2_, ζ_T8_) in 2.6 min by high-performance liquid chromatography (HPLC); **b**–**h** Identification of the selected proteo-specific peptides by high-resolution mass spectrometry (HRMS)

Tryptic digestion of the hemoglobin chains released a series of proteo-specific peptides. Separating these peptides using HPLC followed by identification using HRMS allowed selection of the most informative peptides that corresponded to the respective globin chains (Fig. 1, Supplemental Fig. 1). These were used as candidate biomarkers in our screening assay (Table 2, Supplemental Table 5–11). For abnormal Hb disorders, we selected peptides containing the corresponding mutant amino acid residue (Table 2, Supplemental Table 12–18).

**Table 2:** The proteo-specific peptides and the selected mass transitions for screening of thalassemia and abnormal Hb disorders by HPLC–HRMS

Proteo-specific peptides^*a*^	Sequences	Mass transitions (*m*/*z*)	Retention time (min)	Collision energy
αT1	VLSPADK	729.4141/430.2296	1.22	40
αT1 IS	VLSPADK	737.4280/438.2430	1.22	40
αT3	AAWGK	532.2878/461.2507	1.11	36
αT3 IS	AAWGK	540.2800/469.2647	1.11	36
βT1	VHLTPEEK	476.7585/716.3812	1.34	20
βT1 IS	VHLTPEEK	480.7660/724.3958	1.34	16
βT2	SAVTALWGK	466.7636/675.3824	1.67	18
βT2 IS	SAVTALWGK	470.7710/68.3927	1.67	16
γT10	HLDDLK	370.7005/603.3348	1.18	14
γT10 IS	HLDDLK	374.7080/611.3493	1.18	15
δT2	TAVNALWGK	480.2691/787.4461	1.64	16
δT2 IS	TAVNALWGK	484.2700/795.4580	1.64	16
ζT8	VVAAVGDAVK	464.7767/730.4094	1.50	14
ζT8 IS	VVAAVGDAVK	468.7840/738.4230	1.50	14
β_Hb S_T1	VHLTPVEK	461.7715/412.2373	1.41	20
β_Hb E_T3	VNVDEVGGK	458.7404/703.3621	1.39	18
β_Hb D-Los Angeles_ T13	QFTPPVQAAYQK	689.3617/1001.5415	1.55	14
β_Hb G-Coushatta_ T3	VNVDAVGGEALGR	628.8333/659.3471	1.53	20
β_Hb Watford_T1	GHLTPEEK	455.7351/502.2508	0.90	20
α_Hb Nanchang_T3	AAWSK	562.2984/420.2242	1.04	17
α_Hb Hekinan II_T4	VGAHAGEYGADALER	758.3630/1080.4956	1.43	37

^*a*^Stable isotope-labeled proteo-specific peptides were synthesized using Fmoc-Lys (13C, 15N)-OH. The Signal-to-Noise Ratio for all selected peptides was required to be ≥ 10

*m*/*z*, mass to charge ratio, *Hb* hemoglobin, *HPLC–HRMS* high performance liquid chromatography-high resolution mass spectrometry

### Clinical value of globin ratios

**Fig. 2:**
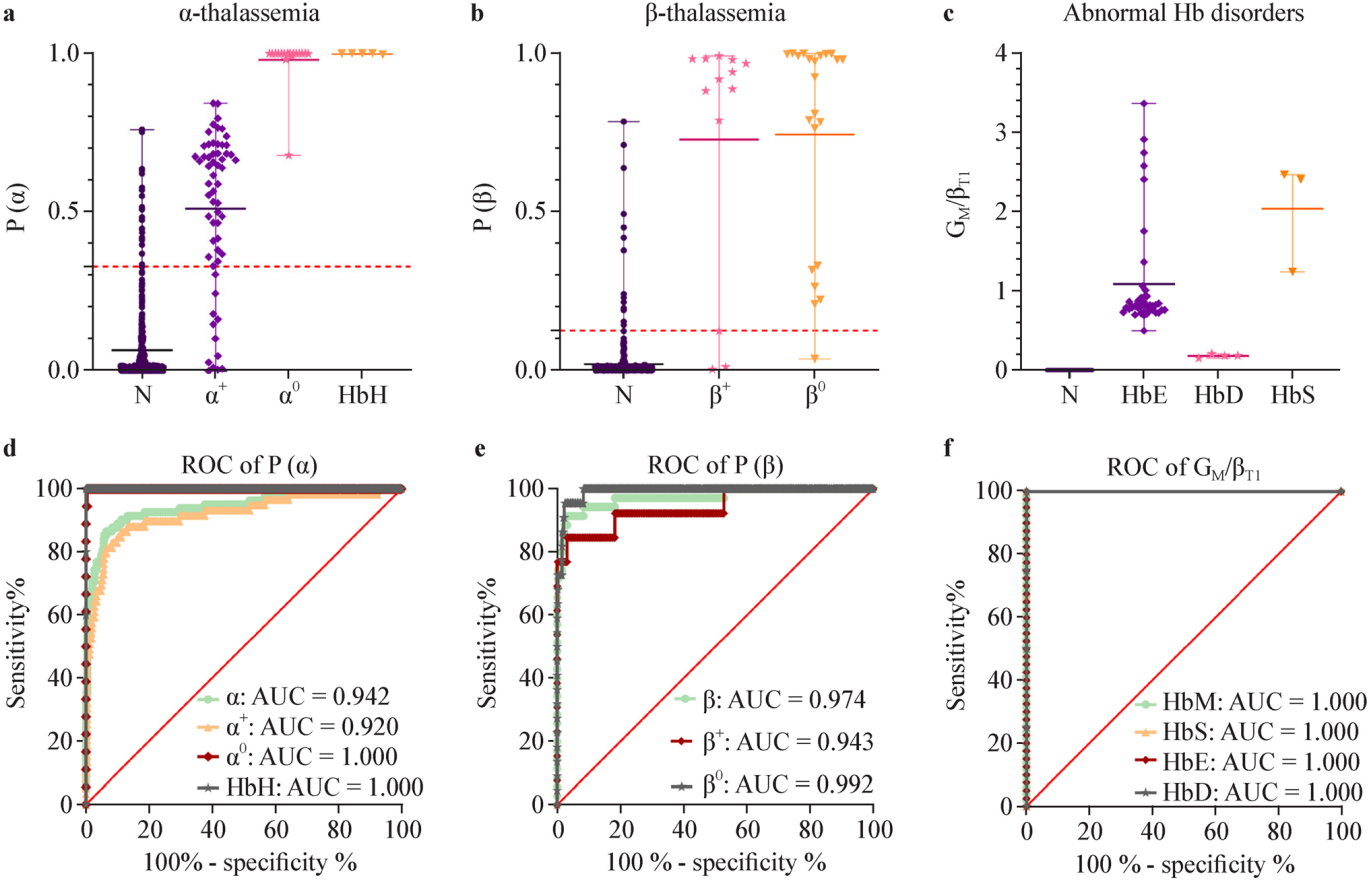
Distribution and receiver operating characteristic (ROC) analysis of the calculated and predicted globin ratios in the groups studied. **a** Distribution of *P*(α) in normal controls (*N*), α^+^, α^0^ and HbH patients. *P*(α) represents the predicted probability of α-thalassemia generated by the predictive model; **b** Distribution of *P*(β) in normal controls (*N*), β^+^and β^0^ patients. *P*(β) represents the predicted probability of β-thalassemia generated by the prediction model; **c** Distribution of the calculated *G*_M_/β ratios in normal controls (*N*) and patients with abnormal Hb disorders (Hb E, Hb D and Hb S); **d**–**f** ROC analysis showing the clinical value of *P*(α), *P*(β) and the *G*_M_/β ratio, when used for the screening of α-thalassemia, β-thalassemia, and abnormal Hb disorders respectively. Hb M is used as a general term for abnormal Hb disorders, including Hb E, Hb D and Hb S

Globin chain ratios were calculated using Eqs. (F1–F3). Mean globin ratios were compared using an unpaired *t*-test in control newborns and patients (Supplemental Fig. 2). ROC curve analysis highlights the clinical value of globin ratios (Supplemental Table 19 and 20). Control individuals and α-thalassemia patients displayed different distributions of ratios α_T1_/β_T1_, α_T1_/β_T2_, γ_T10_/α_T3_, and γ_T10_/β_T1_ (*P* < 0.0001). β-thalassemia patients and control individuals had differentially distributed δ_T2_/β_T2_ ratios (*P* < 0.0001). In patients with Hb S, Hb E, Hb D-Los Angeles (Hb D), Hb G-Coushatta (Hb G), Hb Watford, Hb Nanchang and Hb Hekinan II, the mutated peptide *G*_M_ was easily detected and the *G*_M_/β ratio was effective in disease screening (Fig. 2).

### Optimized screening cut-offs for hemoglobinopathies in newborns

To precisely differentiate hemoglobinopathies a combined dual-indicator test was conducted to enhance the sensitivity and specificity of screening. A prediction model was constructed using binary logistic regression for α-thalassemia using α_T1_/β_T1_ and ζ_T8_/β_T2_:

$$P(\alpha )=\frac{\text{EXP}(30.444*\zeta \text{T}8/\upbeta \text{T}2-1.030*\upalpha \text{T}1/\upbeta \text{T}1+1.771) }{ 1+\text{EXP}(30.444*\zeta \text{T}8/\upbeta \text{T}2-1.030*\upalpha \text{T}1/\upbeta \text{T}1+1.771) }$$

For β-thalassemia, the prediction model using α_T3_/β_T1_ and δ_T2_/β_T2_ was constructed:

$$P(\beta )=\frac{\text{EXP}(105.132*\delta \text{T}2/\beta \text{T}2+0.388*\alpha \text{T}3/\beta \text{T}1-13.195) }{1+\text{EXP}(105.132*\delta \text{T}2/\beta \text{T}2+0.388*\alpha \text{T}3/\beta \text{T}1-13.195)}$$

For each sample, prediction values were calculated and ROC curve analysis was used to determine screening cut-offs. Finally, optimized screening cut-offs for α- and β-thalassemia and abnormal Hb disorders were determined (Table 1).

### Validation of the method

HPLC–HRMS methodology has a sampling and analysis procedure time of 2.6 minutes and can process at least 192 samples per batch costing less than $1 per test. The LLOQ for the proteo-specific peptides was substantially below their actual blood concentration (0.50–60.00 μg/L) (Supplemental Table 21). Inter assay coefficient of variation (CV) (1.53%–14.67%) and intra assay CV (2.78%–14.63%) for the selected peptides was evaluated at three different concentrations (low, medium, and high) (Supplemental Table 22). The trypsin-digested solution remained stable for up to three days at 4 °C prior to sampling. Furthermore, the DBS samples stored under different conditions (RT, 4 °C, and − 20 °C) remained stable for up to one month in the non-digested state (Supplemental Fig. 3). Matrix effects were below 8.30% and carryover rates ranged from 0.20% to 9.99% (Supplemental Table 23 and 24). Extraction recoveries of peptides at low concentration varied between 79.93% and 116.47%, while those at medium and high concentrations varied between 90.15% and 112.94% (Supplemental Table 25).

### Clinical performance of the HPLC–HRMS method

Sebia-CE screening identified 138 positive cases from Group II, while HPLC–HRMS identified 178 positive cases. All cases exhibiting indications of disease were confirmed using NGS. We finally identified 64 cases of α-thalassemia, 40 β-thalassemia, two cases of α- and β-thalassemia and two abnormal Hb disorders (Tables 3 and 4). To evaluate the false-negative rate of the two methods, we performed NGS retesting on 152 randomly selected negative cases, with no additional positive cases identified. For α-thalassemia screening, Sebia-CE had the same sensitivity (68.75%) and a slightly higher specificity than HPLC–HRMS (86.62% vs. 79.93%). The HPLC–HRMS method was more sensitive for αα/--^SEA^ (100.00% vs. 74.07%). For β-thalassemia screening, HPLC–HRMS demonstrated satisfactory performance with a much higher screening sensitivity (97.50% vs. 40.00%). Only HPLC–HRMS identified two cases as heterozygous for α and β variants (ααα^anti3.7^/αα & β^N^/β^IVS-II-654^, HKαα/αα & β^N^/β^Codon 17^) and two cases carrying the Hb D variant.

**Table 3:** Comparison of the clinical performance of HPLC–HRMS and Sebia-CE

Methods		NGS		Total	Sensitivity^a^	Specificity^a^
		+	−			
α-thalassemia						
HPLC–HRMS	+	44	60	104	68.75% (44/64)	79.93% (239/299)
	−	20	239	259		
Sebia-CE	+	44	40	84	68.75% (44/64)	86.62% (259/299)
	−	20	259	279		
β-thalassemia						
HPLC–HRMS	+	39	31	70	97.50% (39/40)	89.63% (268/299)
	−	1	268	269		
Sebia-CE	+	16	33	49	40.00% (16/40)	88.96% (266/299)
	−	24	266	290		
α- and β-thalassemia						
HPLC–HRMS	+	2	0	2	100.00% (2/2)	100.00% (299/299)
	−	0	299	299		
Sebia-CE	+	0	0	0	0.00 (0/2)	100.00% (299/299)
	−	2	299	301		
Abnormal Hb disorders						
HPLC–HRMS	+	2	0	2	100.00% (2/2)	100.00%
	−	0	299	299		(299/299)
Sebia-CE	+	2	3	5	100.00% (2/2)	99.10%
	−	0	296	296		(296/299)

^*a*^The sensitivity and specificity were evaluated based on the cases confirmed by NGS from Group II

*NGS* next-generation sequencing, *HPLC–HRMS* high performance liquid chromatography-high resolution mass spectrometry

**Table 4:** Genotypes of the cases found by HPLC–HRMS and Sebia-CE

Genotypes^a^	Total	Cases detected by HPLC–HRMS (Sensitive)	Cases detected by Sebia-CE (sensitive)
α-thalassemia	64	44 (68.75%)	44 (68.75%)
αα/--^SEA^; β^N^/β^N^	27	27 (100.00%)	20 (74.07%)
αα/-α^3.7^; β^N^/β^N^	19	7 (36.84%)	14 (73.68%)
αα/-α^4.2^; β^N^/β^N^	3	2 (66.67%)	2 (66.67%)
αα/α^WS^α; β^N^/β^N^	1	1 (100.00%)	0 (0.00)
αα/α^CS^α; β^N^/β^N^	5	1 (20.00%)	4 (80.00%)
-α^3.7^/-α^3.7^; β^N^/β^N^	2	0 (0.00)	2 (100.00%)
ααα^anti3.7^/αα; β^N^/β^N^	2	2 (100.00%)	0 (0.00)
ααα^anti4.2^/αα; β^N^/β^N^	4	3 (75.00%)	2 (50.00%)
ααα^anti3.7^/--^SEA^; β^N^/β^N^	1	1 (100.00%)	0 (0.00)
β-thalassemia	40	39 (97.50%)	16 (40.00%)
αα/αα; β^N^/β^Codon 17^	13	13 (100.00%)	5 (38.46%)
αα/αα; β^N^/β^IVS-II-654^	13	13 (100.00%)	5 (38.46%)
αα/αα; β^N^/β^Codons 41/42^	11	10 (90.91%)	6 (54.55%)
αα/αα; β^N^/β^Codons 27/28^	1	1 (100.00%)	0 (0.00)
αα/αα; β^N^/β^Codons 71/72^	1	1 (100.00%)	0 (0.00)
αα/αα; Chinese ^G^γ^+^(^A^γδβ)^0^	1	1 (100.00%)	0 (0.00)
α- and β-thalassemia	2	2 (100.00%)	0 (0.00)
ααα^anti3.7^/αα; β^N^/β^IVS-II-654^	1	1 (100.00%)	0 (0.00)
HKαα/αα; β^N^/β^Codon 17^	1	1 (100.00%)	0 (0.00)
Abnormal Hb disorders	2	2 (100.00%)	2 (100.00%)
Hb D-Los Angeles	2	2 (100.00%)	2 (100.00%)

^a^ HGVS nomenclature: ααα^anti3.7^ = NC_000016.9: g.223300_227103dup. ααα^anti4.2^ = NC_000016.9: g.219817(223755_224074)dup. β^Codons 27/28^ = HBB: c.84_85insC. Chinese ^G^γ^+^(^A^γδβ)^0^ = NC_000011.9: g.5191148_ 5270051del. NC_000016.9: g.219817(223755_ 224074)del. HKαα allele results from a complex rearrangement of the α-globin gene cluster containing both the -α^3.7^ and ααα^anti4.2^ unequal crossover junctions on the same chromosome

*HPLC–HRMS* high performance liquid chromatography-high resolution mass spectrometry

## Discussion

Severity of thalassemia depends on the degree of imbalance in the α-like and β-like globin chains [17, 18]. Silent carriers of α-thalassemia (-α/αα or αα/-α) and those with the α-thalassemia trait (--/αα or -α/-α) are asymptomatic and require no treatment [19]. α-thalassemia intermedia (--/-α, HbH) frequently causes hemolytic anemia and α-thalassemia major (--/--, Hb Bart’s) usually results in fatal hydrops fetalis [20]. β-thalassemia major (β^0^) causes hemolytic anemia, poor growth and skeletal abnormalities during infancy. Affected children rely on regular lifelong blood transfusions [21–23]. β-thalassemia intermedia (β^+^) is less severe than β-thalassemia major and requires episodic blood transfusions. Patients who coinherit Hb S, Hb C, Hb E or Hb D with another pathogenic β-globin chain variant (β^+^ or β^0^) develop abnormal hemoglobin disorders. This could trigger clinical symptoms such as intermittent vaso-occlusive events, chronic hemolysis and varying degrees of anemia, jaundice and growth retardation [24].

In this study, we focused on clinically relevant hemoglobin variants, specifically those known to cause symptomatic disease or confer inheritable risk to offspring. As Hb Bart’s usually results in fatal hydrops fetalis, we analyzed two clinically significant forms of α-thalassemia; α-thalassemia intermedia or HbH disease and α-thalassemia trait, which have two or three α-globin genes deleted or inactivated. For β-thalassemia, we also included two clinically significant forms, β-thalassemia major (β^0^) and β-thalassemia intermedia (β^+^). For each type of thalassemia, we identified proteo-specific peptides, then calculated globin ratios to reflect the degree of imbalance between the α-like and β-like globin chains. Abnormal Hb disorders, including Hb S, Hb E, Hb D, Hb G and some rare types were tested to validate the feasibility of using the proportion of mutated Hb subunits for disease screening.

To precisely differentiate patients, a combined dual-indicator test was used to improve the sensitivity and specificity of screening. Initial evaluation of the clinical value of globin ratios showed that α_T1_/β_T1_ (AUC = 0.927) had the highest diagnostic value for α-thalassemia, while δ_T2_/β_T2_ (AUC = 0.862) had the highest diagnostic performance for β-thalassemia (Supplemental Table 19 and 20). After birth, Hb undergoes a rapid transformation. However, some embryonic globins, such as ζ and γ, are persistently expressed in newborns with thalassemia. The δ globin chain, which composes HbA2 and is recognized as a reliable marker for β-thalassemia screening in children and adults, is expressed at low or undetectable levels in newborns [25, 26]. Therefore, a single screening marker does not meet the demand for high sensitivity and specificity in screening newborns. A prediction model using binary logistic regression for α-thalassemia using α_T1_/β_T1_ and ζ_T8_/β_T2_ (*P*(α) > 0.329, AUC = 0.942) and for β-thalassemia using δ_T2_/β_T2_ and α_T3_/β_T1_ (*P*(β) > 0.126, AUC = 0.974) was constructed. Application of this prediction model to a cohort of 2726 newborns demonstrated its ability to efficiently detect clinically significant forms of α-thalassemia (--^SEA^) and most types of β-thalassemia.

Method assessment showed that this method was reliable in clinical application. Inter and intra assay CV for the proteo-specific peptides measured were below 14.67% and the LLOQs for selected peptides were much lower than their actual concentrations in DBS samples. We observed no appreciable matrix effects or carryover and the method had satisfactory extraction recoveries. Calculated globin ratios were stable (CV < 20%) for at least 30 days when the DBS samples were stored at RT, 4 °C, and − 20 °C. This could be an advantage over top–down MS approaches and traditional methods, as it allows for long-distance transportation of DBS samples for centralized screening.

Evaluation of clinical performance showed that the HPLC–HRMS was superior to the Sebia-CE method. Although they have the same sensitivity (68.8%) for α-thalassemia, the HPLC–HRMS method performed better for --^SEA^, which accounts for majority of symptomatic cases. For β-thalassemia screening, the HPLC–HRMS method was markedly more efficient than Sebia-CE, with a higher sensitivity (97.50%) and a similar specificity (89.63%). HPLC–HRMS detected two cases that were simultaneously heterozygous for α and β variants (ααα^anti3.7^/αα & β^IVS-II-654^/β^N^, and HKαα/αα & β^Codon 17^/β^N^) through the β-thalassemia prediction model; these were missed by Sebia-CE. HPLC–HRMS directly confirmed two cases of Hb D in the validation cohort, with no instances of Hb S or Hb E detected. In addition, this method can be adapted to identify other rare abnormal Hb variants when needed.

The HPLC–HRMS method employs a simple pre-processing procedure: hemoglobin is digested with trypsin, followed by extraction and detection of proteo-specific peptide biomarkers. Quantification is performed using internal standards, eliminating the need for biomarker-specific calibration curves. Sample preparation is carried out in 96-well plates, enabling high-throughput processing of at least 192 samples (two plates) per batch at a cost of less than $1 per test. The method allows use of the same heel-prick DBS samples collected for newborn inherited metabolic disease screening. The entire workflow, from sample collection 48 hours after birth, transportation and laboratory testing to reporting, is completed within one week (under a centralized testing model in China).

There are limitations to this methodology. Prediction requires more patients with different genotypes to generate more efficient cut-offs to improve prediction accuracy. Due to the prospective group size, a limited number of disease types were detected. Future work to substantiate the efficacy of this approach is to enlist a greater number of participants from diverse geographical locations, encompassing a broader range of genotypes and age groups. This would facilitate a more comprehensive screening of hemoglobinopathies in newborns, children, and adults.

In conclusion, we have developed an HPLC–HRMS method capable of efficiently detecting clinically significant forms of hemoglobinopathies in newborns, including α-thalassemia, β-thalassemia and abnormal Hb disorders. This newly developed method is rapid, high-throughput, cost-effective and could serve as a first-line screening test in newborns.

## Supplementary Information

Below is the link to the electronic supplementary material.

Supplementary file1 (PDF 1310 KB)

## Data Availability

The data set underlying the findings of this paper is available on request. De-identified data will be made available to qualified researchers upon reasonable request to the corresponding author (chaowenyu@hospital.cqmu.edu.cn), subject to institutional data transfer agreements and ethical approvals.
