# Supplementary material for: Development of a low-cost and high-throughput LC–MS method for newborn screening of thalassemia and abnormal hemoglobin disorders
Source: World J Pediatr. 2025 Sep 1;21(9):889–901. doi: 10.1007/s12519-025-00962-y (PMC12433368; doi:10.1007/s12519-025-00962-y)
Supplement: Supplementary file 1 — Supplementary file1 (PDF 1310 KB) [file 12519_2025_962_MOESM1_ESM.pdf]

Supplemental materials

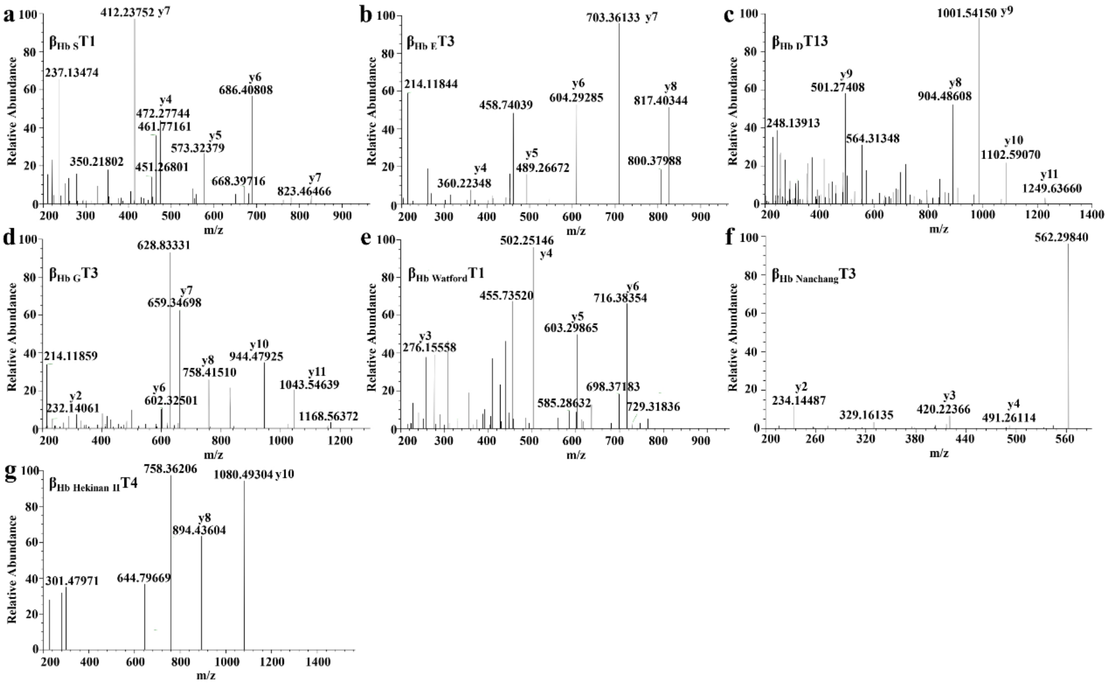

Supplemental **Figure 1**. Identification of the selected proteo-specific peptides generated from abnormal Hb disorders by HRMS. Representative mass spectrometry spectra of the selected proteo-specific peptides are shown: (a) $\beta_{Hb\ S}$T1, (b) $\beta_{Hb\ E}$T3, (c) $\beta_{Hb\ D}$T13, (d) $\beta_{Hb\ G\text{-}Coushatta}$T3, (e) $\beta_{Hb\ Watford}$T1, (f) $\beta_{Hb\ Nanchang}$T3, and (g) $\beta_{Hb\ Hekinan\ II}$T4.

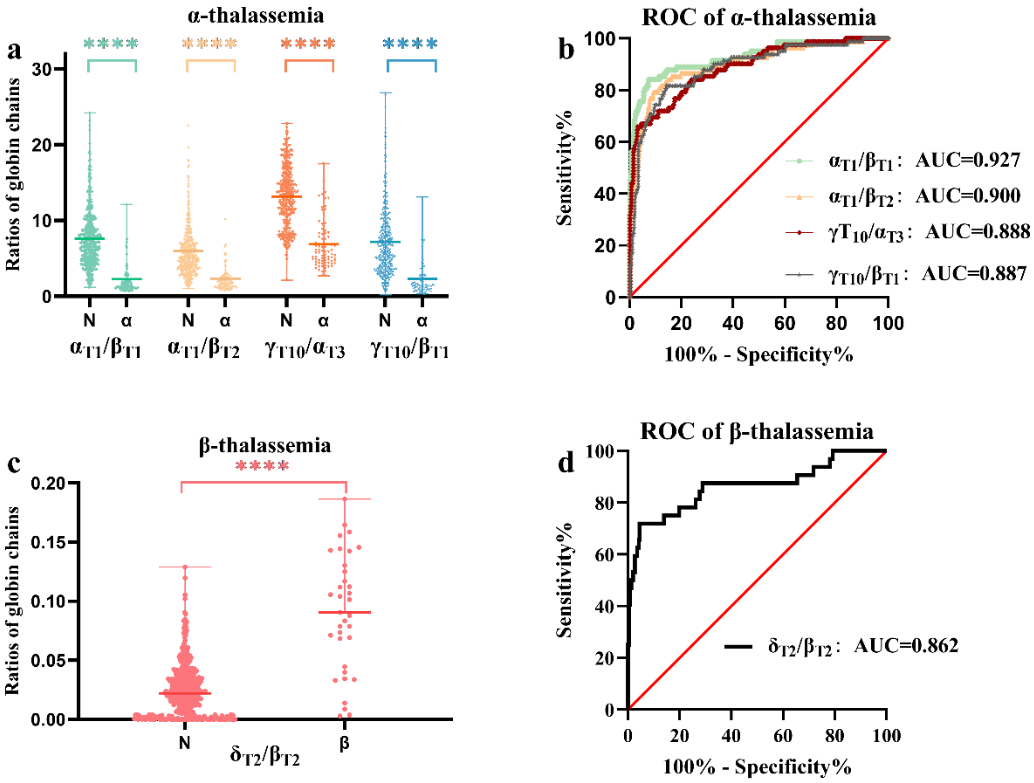

Supplemental **Figure 2**. The distribution of the calculated globin ratios in the studied groups and the ROC analysis. (a) Distribution of the calculated α_{T1}/β_{T1}, α_{T1}/β_{T2}, γ_{T10}/α_{T3} and γ_{T10}/β_{T1} ratios in normal controls (N) and α-thalassemia patients (α). (b) The ROC analysis to show the clinical value of the α_{T1}/β_{T1}, α_{T1}/β_{T2}, γ_{T10}/α_{T3} and γ_{T10}/β_{T1} ratios used for α-thalassemia screening. (c) Distribution of the calculated δ_{T2}/β_{T2} ratios in normal controls (N) and β-thalassemia patients (β). (d) The ROC analysis to show the clinical value of the δ_{T2}/β_{T2} ratio used for β-thalassemia screening. Statistical significance currently used was defined as $p<0.0001$ (****).

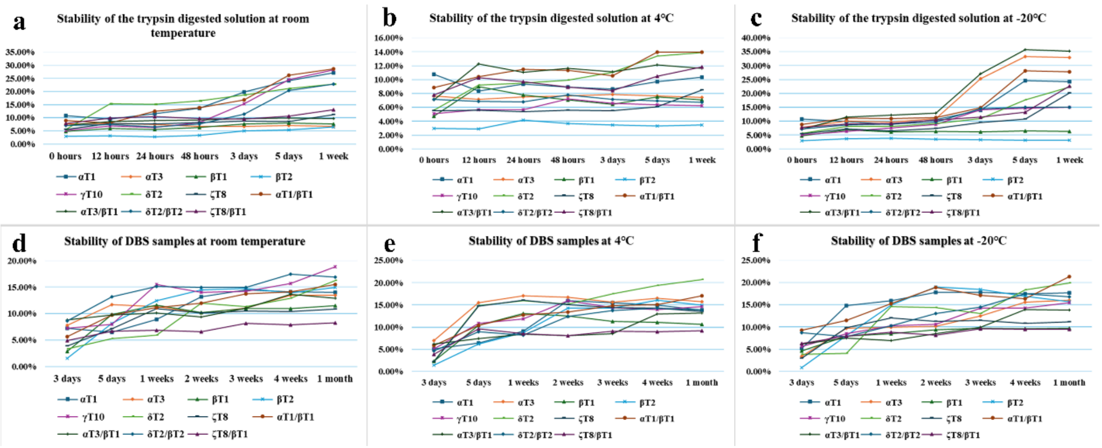

Supplemental **Figure 3**. The stability of the selected proteo-specific peptides tested by HPLC-HRMS. a-c: Stability of the final solution prepared for sampling within one week. d-f: Stability of the DBS samples stored at different conditions (Room temperature, 4°C, -20°C).

Supplemental **Table 1**. The gradient flow rate used for peptide separation by HPLC

Time (min)	Flow (ml/min)	%A	%B	Curve
0.000	0.500	92.0	8.0	5
0.350	0.500	73.0	27.0	5
1.200	0.500	47.0	53.0	5
1.500	0.500	6.0	94.0	5
2.000	0.500	5.0	95.0	5
2.000	0.500	95.0	5.0	5
2.600	Stop Run			

A: Deionized water +formic acid (0.1%).
B: Acetonitrile +formic acid (0.1%).

Supplemental **Table 2**. The ion source parameters set for mass spectrometry analysis

HESI source parameters	Values
Sheath gas flow rate (arb)	55
Aux gas flow rate (arb)	15
Sweep gas flow rate (arb)	0
Spray voltage (KV)	3.5
Spray current (μA)	0
Capillary Temp (℃)	320
S-lens RF level	50
Aux gas heater temp (℃)	400

Supplemental **Table 3**. The parameters of Trace Finder software used for peak assignment

Items	Values
Detection type	Single-Detected
Sensitivity	ICIS
Detection method	Nearest
Peak Threshold Type	Area
RT Smoothing	5
Area Noise Facto	1000
Peak Noise Factor	10
Baseline Window	80
Min Peak Height(S/N)	3.0
Mass tolerance	50ppm

Supplemental **Table 4.** The recommended decision-making rules of the Sebia CE method

Thalassemia type	Hb Bart's	Hb E	Hb A fraction	Hb A2/Hb A ratio
α-thalassemia	> 0	-	-	-
	-	-	≤ 7.3%	-
	-	-	7.4%-9.5%	> 0
	-	-	9.6%-11.5%	> 0.008
β-thalassemia	-	-	11.6%-15.0%	> 0.015
	-	-	15.1%-20.0%	> 0.020
	-	-	> 20.0%	> 0.025
	-	> 0	-	-

Supplemental **Table 5.** Fragments generated by collision-induced dissociation from α_{T1}

SN	Mass (+1)	Mass (+2)	Fragment Sequences (b)	Fragment Sequences (y)	Mass (+1)	Mass (+2)	SN
b1	118.0863	59.5468	V	LSPADK	630.3457	315.6765	y6
b2	231.1703	116.0888	VL	SPADK	517.2617	259.1345	y5
b3	318.2024	159.6048	VLS	PADK	430.2296	215.6185	y4
b4	415.2551	208.1312	VLSP	ADK	333.1769	167.0921	y3
b5	486.2922	243.6498	VLSPA	DK	262.1398	131.5735	y2
b6	601.3192	301.1632	VLSPAD	K	147.1128	74.0600	y1

SN, Sequence Number.

Supplemental **Table 6.** Fragments generated by collision-induced dissociation from α_{T3}

SN	Mass (+1)	Mass (+2)	Fragment Sequences (b)	Fragment Sequences (y)	Mass (+1)	Mass (+2)	SN
b1	90.0550	45.5311	A	AWGK	461.2507	231.1290	y4
b2	161.0921	81.0497	AA	WGK	390.2136	195.6104	y3
b3	347.1714	174.0893	AAW	GK	204.1343	102.5708	y2
b4	404.1929	202.6001	AAWG	K	147.1128	74.0600	y1

SN, Sequence Number.

Supplemental **Table 7.** Fragments generated by collision-induced dissociation from β_{T1}

SN	Mass (+1)	Mass (+2)	Fragment Sequences (b)	Fragment Sequences (y)	Mass (+1)	Mass (+2)	SN
b1	118.0863	59.5468	V	HLTPEEK	853.4414	427.2243	y7
b2	255.1452	128.0762	VH	LTPEEK	716.3825	358.6949	y6
b3	368.2292	184.6183	VHL	TPEEK	603.2984	302.1529	y5
b4	469.2769	235.1421	VHLT	PEEK	502.2508	251.6290	y4
b5	566.3297	283.6685	VHLTP	EEK	405.1980	203.1026	y3
b6	695.3723	348.1898	VHLTPE	EK	276.1554	138.5813	y2
b7	824.4149	412.7111	VHLTPEE	K	147.1128	74.0600	y1

SN, Sequence Number.

Supplemental **Table 8.** Fragments generated by collision-induced dissociation from β_{T2}

SN	Mass (+1)	Mass (+2)	Fragment Sequences (b)	Fragment Sequences (y)	Mass (+1)	Mass (+2)	SN
b1	106.0499	53.5286	S	AVTALWGK	845.4880	423.2476	y8
b2	177.0870	89.0471	SA	VTALWGK	774.4509	387.7291	y7
b3	276.1554	138.5813	SAV	TALWGK	675.3824	338.1949	y6
b4	377.2031	189.1052	SAVT	ALWGK	574.3348	287.6710	y5
b5	448.2402	224.6237	SAVTA	LWGK	503.2976	252.1525	y4
b6	561.3243	281.1658	SAVTAL	WGK	390.2136	195.6104	y3
b7	747.4036	374.2054	SAVTALW	GK	204.1343	102.5708	y2
b8	804.4250	402.7162	SAVTALWG	K	147.1128	74.0600	y1

SN, Sequence Number.

Supplemental **Table 9.** Fragments generated by collision-induced dissociation from γ_{T10}

SN	Mass (+1)	Mass (+2)	Fragment Sequences (b)	Fragment Sequences (y)	Mass (+1)	Mass (+2)	SN
b1	156.0768	78.5420	H	LDDLK	603.3348	302.1711	y5
b2	269.1608	135.0841	HL	DDLK	490.2508	245.6290	y4
b3	384.1878	192.5975	HLD	DLK	375.2238	188.1155	y3
b4	499.2147	250.1110	HLDD	LK	260.1969	130.6021	y2
b5	612.2988	306.6530	HLDDL	K	147.1128	74.0600	y1

SN, Sequence Number.

Supplemental **Table 10**. Fragments generated by collision-induced dissociation from δ_{T2}

SN	Mass (+1)	Mass (+2)	Fragment Sequences (b)	Fragment Sequences (y)	Mass (+1)	Mass (+2)	SN
b1	120.0655	60.5364	T	AVNALWGK	858.4832	429.7453	y8
b2	191.1026	96.0550	TA	VNALWGK	787.4461	394.2267	y7
b3	290.1711	145.5892	TAV	NALWGK	688.3777	344.6925	y6
b4	404.2140	202.6106	TAVN	ALWGK	574.3348	287.6710	y5
b5	475.2511	238.1292	TAVNA	LWGK	503.2976	252.1525	y4

b6	588.3352	294.6712	TAVNAL	WGK	390.2136	195.6104	y3
b7	774.4145	387.7109	TAVNALW	GK	204.1343	102.5708	y2
b8	831.4359	416.2216	TAVNALWG	K	147.1128	74.0600	y1

SN, Sequence Number.

Supplemental **Table 11.** Fragments generated by collision-induced dissociation from ζ_{T8}

SN	Mass (+1)	Mass (+2)	Fragment Sequences (b)	Fragment Sequences (y)	Mass (+1)	Mass (+2)	SN
b1	118.0863	59.5468	V	VAAVGDAVK	829.4778	415.2425	y9
b2	217.1547	109.0810	VV	AAVGDAVK	730.4094	365.7083	y8
b3	288.1918	144.5995	VVA	AVGDAVK	659.3723	330.1898	y7
b4	359.2289	180.1181	VVAA	VGDAVK	588.3352	294.6712	y6
b5	458.2973	229.6523	VVAAV	GDAVK	489.2667	245.1370	y5
b6	515.3188	258.1630	VVAAVG	DAVK	432.2453	216.6263	y4
b7	630.3457	315.6765	VVAAVGD	AVK	317.2183	159.1128	y3
b8	701.3828	351.1951	VVAAVGDA	VK	246.1812	123.5943	y2
b9	800.4512	400.7293	VVAAVGDAV	K	147.1128	74.0600	y1

SN, Sequence Number.

Supplemental **Table 12.** Fragments generated by collision-induced dissociation from $\beta_{Hb\ S}$T1

SN	Mass (+1)	Mass (+2)	Fragment Sequences (b)	Fragment Sequences (y)	Mass (+1)	Mass (+2)	SN
b1	118.0863	59.5468	V	HLTPVEK	823.4672	412.2373	y7
b2	255.1452	128.0762	VH	LTPVEK	686.4083	343.7078	y6
b3	368.2292	184.6183	VHL	TPVEK	573.3243	287.1658	y5
b4	469.2769	235.1421	VHLT	PVEK	472.2766	236.6419	y4
b5	566.3297	283.6685	VHLTP	VEK	375.2238	188.1155	y3
b6	665.3981	333.2027	VHLTPV	EK	276.1554	138.5813	y2
b7	794.4407	397.7240	VHLTPVE	K	147.1128	74.0600	y1

SN, Sequence Number.

Supplemental **Table 13.** Fragments generated by collision-induced dissociation from $\beta_{Hb\ E}$T3

SN	Mass (+1)	Mass (+2)	Fragment Sequences (b)	Fragment Sequences (y)	Mass (+1)	Mass (+2)	SN
b1	118.0863	59.5468	V	NVDEVGGK	817.4050	409.2062	y8
b2	232.1292	116.5682	VN	VDEVGGK	703.3621	352.1847	y7
b3	331.1976	166.1024	VNV	DEVGGK	604.2937	302.6505	y6
b4	446.2245	223.6159	VNVD	EVGGK	489.2667	245.1370	y5
b5	575.2671	288.1372	VNVDE	VGGK	360.2242	180.6157	y4
b6	674.3356	337.6714	VNVDEV	GGK	261.1557	131.0815	y3
b7	731.3570	366.1821	VNVDEVG	GK	204.1343	102.5708	y2
b8	788.3785	394.6929	VNVDEVGG	K	147.1128	74.0600	y1

SN, Sequence Number.

Supplemental **Table 14.** Fragments generated by collision-induced dissociation from $\beta_{Hb\ D\text{-}Los\ Angeles}$T13

SN	Mass (+1)	Mass (+2)	Fragment Sequences (b)	Fragment Sequences (y)	Mass (+1)	Mass (+2)	SN
b1	147.0764	74.0419	Q	FTPPVQAAYQK	1249.6576	625.3324	y11
b2	294.1448	147.5761	QF	TPPVQAAYQK	1102.5891	551.7982	y10
b3	395.1925	198.0999	QFT	PPVQAAYQK	1001.5415	501.2744	y9
b4	492.2453	246.6263	QFTP	PVQAAYQK	904.4887	452.7480	y8
b5	589.2980	295.1527	QFTPP	VQAAYQK	807.4359	404.2216	y7
b6	688.3665	344.6869	QFTPPV	QAAYQK	708.3675	354.6874	y6
b7	816.4250	408.7162	QFTPPVQ	AAYQK	580.3089	290.6581	y5
b8	887.4621	444.2347	QFTPPVQA	AYQK	509.2718	255.1396	y4
b9	958.4993	479.7533	QFTPPVQAA	YQK	438.2347	219.6210	y3
b10	1121.5626	561.2849	QFTPPVQAAY	QK	275.1714	138.0893	y2
b11	1249.6212	625.3142	QFTPPVQAAYQ	K	147.1128	74.0600	y1

SN, Sequence Number.

Supplemental **Table 15.** Fragments generated by collision-induced dissociation from $\beta_{Hb\ G\text{-}Coushatta}$T3

SN	Mass (+1)	Mass (+2)	Fragment Sequences (b)	Fragment Sequences (y)	Mass (+1)	Mass (+2)	SN
b1	118.0863	59.5468	V	NVDAVGGEALGR	1157.5909	579.2991	y12
b2	232.1292	116.5682	VN	VDAVGGEALGR	1043.5480	522.2776	y11
b3	331.1976	166.1024	VNV	DAVGGEALGR	944.4796	472.7434	y10
b4	446.2245	223.6159	VNVD	AVGGEALGR	829.4526	415.2300	y9
b5	517.2617	259.1345	VNVDA	VGGEALGR	758.4155	379.7114	y8
b6	616.3301	308.6687	VNVDAV	GGEALGR	659.3471	330.1772	y7
b7	673.3515	337.1794	VNVDAVG	GEALGR	602.3257	301.6665	y6
b8	730.3730	365.6901	VNVDAVGG	EALGR	545.3042	273.1557	y5
b9	859.4156	430.2114	VNVDAVGGE	ALGR	416.2616	208.6344	y4
b10	930.4527	465.7300	VNVDAVGGEA	LGR	345.2245	173.1159	y3
b11	1043.5368	522.2720	VNVDAVGGEAL	GR	232.1404	116.5739	y2
b12	1100.5582	550.7828	VNVDAVGGEALG	R	175.1190	88.0631	y1

SN, Sequence Number.

Supplemental **Table 16.** Fragments generated by collision-induced dissociation from $\beta_{Hb\ Watford}$T1

SN	Mass (+1)	Mass (+2)	Fragment Sequences (b)	Fragment Sequences (y)	Mass (+1)	Mass (+2)	SN
b1	76.0393	38.5233	G	HLTPEEK	853.4414	427.2243	y7
b2	213.0982	107.0528	GH	LTPEEK	716.3825	358.6949	y6
b3	326.1823	163.5948	GHL	TPEEK	603.2984	302.1529	y5
b4	427.2300	214.1186	GHLT	PEEK	502.2508	251.6290	y4
b5	524.2827	262.6450	GHLTP	EEK	405.1980	203.1026	y3

b6	653.3253	327.1663	GHLTPE	EK	276.1554	138.5813	y2
b7	782.3679	391.6876	GHLTPEE	K	147.1128	74.0600	y1

SN, Sequence Number.

Supplemental **Table 17.** Fragments generated by collision-induced dissociation from $\alpha_{Hb\ Nanchang}$T3

SN	Mass (+1)	Mass (+2)	Fragment Sequences (b)	Fragment Sequences (y)	Mass (+1)	Mass (+2)	SN
b1	90.0550	45.5311	A	AWSK	491.2613	246.1343	y4
b2	161.0921	81.0497	AA	WSK	420.2242	210.6157	y3
b3	347.1714	174.0893	AAW	SK	234.1448	117.5761	y2
b4	434.2034	217.6053	AAWS	K	147.1128	74.0600	y1

SN, Sequence Number.

Supplemental **Table 18.** Fragments generated by collision-induced dissociation from $\alpha_{Hb\ Hekinan\ II}$T4

SN	Mass (+1)	Mass (+2)	Fragment Sequences (b)	Fragment Sequences (y)	Mass (+1)	Mass (+2)	SN
b1	118.0863	59.5468	V	GAHAGEYGADALER	1416.6502	708.8288	y14
b2	175.1077	88.0575	VG	AHAGEYGADALER	1359.6288	680.3180	y13
b3	246.1448	123.5761	VGA	HAGEYGADALER	1288.5917	644.7995	y12
b4	383.2037	192.1055	VGAH	AGEYGADALER	1151.5327	576.2700	y11
b5	454.2409	227.6241	VGAHA	GEYGADALER	1080.4956	540.7515	y10
b6	511.2623	256.1348	VGAHAG	EYGADALER	1023.4742	512.2407	y9
b7	640.3049	320.6561	VGAHAGE	YGADALER	894.4316	447.7194	y8
b8	803.3682	402.1878	VGAHAGEY	GADALER	731.3682	366.1878	y7
b9	860.3897	430.6985	VGAHAGEYG	ADALER	674.3468	337.6770	y6
b10	931.4268	466.2171	VGAHAGEYGA	DALER	603.3097	302.1585	y5
b11	1046.4538	523.7305	VGAHAGEYGAD	ALER	488.2827	244.6450	y4
b12	1117.4909	559.2491	VGAHAGEYGADA	LER	417.2456	209.1264	y3
b13	1230.5749	615.7911	VGAHAGEYGADAL	ER	304.1616	152.5844	y2
b14	1359.6175	680.3124	VGAHAGEYGADALE	R	175.1190	88.0631	y1

SN, Sequence Number.

Supplemental **Table 19.** Evaluation of the clinical value of the ratio between globin peptides in different types of α-thalassemia

Ratio between globin peptides	α-thalassemia (N = 79)	The AUC values generated by ROC analysis [a]									
		$\alpha\alpha/--^{SEA}$ (N = 16)	$\alpha\alpha/-\alpha^{3.7}$ (N = 26)	$\alpha\alpha/-\alpha^{4.2}$ (N = 15)	$\alpha\alpha/\alpha^{CS}\alpha$ (N = 9)	$\alpha\alpha/\alpha^{QS}\alpha$ (N = 1)	$\alpha\alpha/\alpha^{WS}\alpha$ (N = 5)	$\alpha\alpha/\alpha^{Groene\ Hart}\alpha$ (N = 1)	$--^{SEA}/-\alpha^{3.7}$ (N = 2)	$--^{SEA}/-\alpha^{4.2}$ (N = 3)	$-\alpha^{3.7}/-\alpha^{3.7}$ (N = 1)
α_{T1}/β_{T1}	**0.927**	0.985	0.809	**0.983**	0.991	**0.998**	**0.997**	0.792	0.997	0.994	**0.993**
α_{T1}/β_{T2}	0.900	0.965	0.788	0.963	0.971	**0.998**	0.991	0.957	0.980	0.987	0.955
α_{T3}/β_{T1}	0.826	0.904	0.715	0.860	0.910	0.896	0.706	**0.993**	0.966	0.945	0.892
α_{T3}/β_{T2}	0.638	0.651	0.585	0.663	0.715	0.801	0.844	0.989	0.773	0.789	0.582
δ_{T2}/α_{T1}	0.825	0.853	0.709	0.921	0.947	0.953	0.518	0.968	0.699	0.625	0.878
δ_{T2}/α_{T3}	0.693	0.644	0.626	0.787	0.820	0.686	0.995	0.508	0.591	0.502	0.625
δ_{T2}/β_{T1}	0.587	0.548	0.559	0.692	0.706	0.501	0.917	0.637	0.582	0.686	0.571
δ_{T2}/β_{T2}	0.712	0.621	0.647	0.834	0.864	0.598	0.962	0.926	0.500	0.545	0.736
γ_{T10}/α_{T1}	0.552	0.791	0.554	0.531	0.656	0.849	0.922	0.935	0.989	1.000	0.916
γ_{T10}/α_{T3}	0.888	0.911	**0.817**	0.945	**0.995**	0.993	0.783	0.946	0.796	0.582	0.973
γ_{T10}/β_{T1}	0.887	0.939	0.781	0.934	0.978	0.966	0.557	0.939	0.953	0.865	0.946
γ_{T10}/β_{T2}	0.837	0.887	0.752	0.881	0.951	0.968	0.869	0.939	0.867	0.735	0.810
ζ_{T8}/α_{T1}	0.674	**1.000**	0.675	0.502	0.591	0.506	0.713	0.928	**1.000**	**1.000**	0.822
ζ_{T8}/α_{T3}	0.527	**1.000**	0.525	0.742	0.835	0.813	0.651	0.905	**1.000**	**1.000**	0.625
ζ_{T8}/β_{T1}	0.535	0.978	0.569	0.802	0.913	0.894	0.757	0.894	0.991	0.978	0.743
ζ_{T8}/β_{T2}	0.513	0.992	0.516	0.735	0.868	0.856	0.504	0.810	0.995	0.992	0.551
δ_{T2}/ζ_{T8}	0.589	0.775	0.582	0.802	0.897	0.808	0.962	0.799	0.819	0.822	0.632
ζ_{T8}/γ_{T10}	0.673	**1.000**	0.681	0.535	0.544	0.587	0.915	0.932	**1.000**	**1.000**	0.686

[a] AUC, area under the curve; ROC analysis, the receiver operating characteristic curve analysis; The highlighted values represent the maximum AUC corresponding to the ratio between globin peptides detected in patients with different types of thalassemia.

Supplemental **Table 20.** Evaluation of the clinical value of the ratio between globin peptides in different types of β-thalassemia

Ratio between globin peptides	β-thalassemia (N = 32)	The AUC values generated by ROC analysis [a]									α- and β-thalassemia (N = 3)
		$\beta^N/\beta^{Codon\ 17}$ (N = 3)	$\beta^N/\beta^{Codons\ 41/42}$ (N = 16)	$\beta^N/\beta^{Codons\ 71/72}$ (N = 2)	$\beta^N/\beta^{IVS\text{-}II\text{-}654}$ (N = 8)	$\beta^N/\beta^{Codon\ 43}$ (N = 1)	$\beta^N/\beta^{3'UTR\ +129\ (T>A)}$ (N = 2)	$\alpha\alpha/\text{-}\alpha^{3.7}$; β^N/β^{-28} (N = 1)	$\alpha\alpha/\alpha^{WS}\alpha$; $\beta^N/\beta^{IVS\text{-}II\text{-}654}$ (N = 1)	$\alpha\alpha/\text{--}^{SEA}$; β^N/β^{-28} (N = 1)	
α_{T1}/β_{T1}	0.702	0.669	0.747	0.500	0.694	0.973	0.519	0.982	0.885	0.998	0.955
α_{T1}/β_{T2}	0.582	0.602	0.607	0.511	0.534	0.962	0.554	0.962	0.673	0.957	0.749
α_{T3}/β_{T1}	0.599	0.654	0.571	0.530	0.678	0.587	0.590	0.946	0.736	0.971	0.727
α_{T3}/β_{T2}	0.785	0.769	0.797	0.533	**0.891**	0.632	0.611	0.799	**0.995**	0.664	0.511
δ_{T2}/α_{T1}	0.802	0.849	0.843	0.870	0.739	0.991	0.505	0.991	0.883	0.998	0.957
δ_{T2}/α_{T3}	0.710	0.795	0.734	0.880	0.617	0.912	0.517	0.973	0.643	0.971	0.862
δ_{T2}/β_{T1}	0.831	**0.950**	0.867	**0.979**	0.743	0.991	0.503	0.993	0.847	0.962	0.934
δ_{T2}/β_{T2}	**0.862**	0.904	**0.904**	0.973	0.808	**0.995**	0.502	**0.998**	0.993	1.000	**0.997**
γ_{T10}/α_{T1}	0.644	0.792	0.614	0.673	0.640	0.982	0.517	0.761	0.849	0.641	0.518
γ_{T10}/α_{T3}	0.796	0.695	0.818	0.568	0.889	0.971	0.677	0.984	0.971	0.984	0.980
γ_{T10}/β_{T1}	0.627	0.503	0.675	0.569	0.623	0.860	0.518	0.966	0.716	0.982	0.888
γ_{T10}/β_{T2}	0.501	0.588	0.533	0.573	0.555	0.781	0.502	0.950	0.772	0.921	0.700
ζ_{T8}/α_{T1}	0.551	0.655	0.509	0.717	0.823	0.907	0.658	0.761	0.722	**1.000**	0.506
ζ_{T8}/α_{T3}	0.631	0.809	0.691	0.745	0.592	0.576	**0.690**	0.878	0.903	**1.000**	0.594
ζ_{T8}/β_{T1}	0.578	0.716	0.646	0.699	0.650	0.551	0.620	0.930	0.837	0.937	0.610
ζ_{T8}/β_{T2}	0.509	0.691	0.567	0.708	0.745	0.524	0.605	0.905	0.616	0.986	0.512
δ_{T2}/ζ_{T8}	0.715	0.857	0.770	0.866	0.547	0.804	0.538	0.977	0.856	0.564	0.756
ζ_{T8}/γ_{T10}	0.520	0.762	0.531	0.770	0.790	0.777	0.570	0.722	0.781	**1.000**	0.501

[a] AUC, area under the curve; ROC analysis, the receiver operating characteristic curve analysis; The highlighted values represent the maximum AUC corresponding to the ratio between globin peptides detected in patients with different types of thalassemia.

Supplemental **Table 21.** The lower limits of quantitation (LLOQ) for the selected peptides detected by HPLC-HRMS [a]

Peptides	S1		S2		S3	
	Concentration (μg/L)	CV	LLOQ (μg/L)	CV	Concentration (μg/L)	CV
αT1	40.00	41.48%	**50.00**	20.24%	60.00	17.22%
αT3	50.00	30.11%	**60.00**	15.13%	70.00	12.46%
βT1	15.00	24.33%	**20.00**	19.07%	25.00	13.74%
βT2	1.00	41.76%	**2.00**	18.34%	5.00	9.55%
δT2	20.00	28.18%	**25.00**	19.34%	30.00	18.41%
γT10	5.00	50.89%	**10.00**	18.63%	20.00	10.81%
ζT8	0.20	32.94%	**0.50**	10.40%	1.00	6.64%

[a] The standard peptides were dissolved by trypsin digested serum to different concentrations. For each concentration level of peptides, 15 repeats were tested to evaluate the coefficient of variation (CV) of the measurements. The measured concentration with the CV close to 20% was defined as the LLOQ for the selected peptides.

Supplemental **Table 22.** Inter and intra assay of the HPLC-HRMS method for Hb-specific peptides detection [a]

Peptides	Concentrations of QC samples	Detection precision (CV)	
		Inter assay (n=5)	intra assay (n=18)
αT1	Low (0.100 mg/L)	10.38%	10.80%
	Medium (1.000 mg/L)	5.55%	7.98%
	High (4.000 mg/L)	3.59%	5.98%
αT3	Low (0.500 mg/L)	14.67%	14.63%
	Medium (1.000 mg/L)	11.64%	10.70%
	High (3.000 mg/L)	4.56%	6.45%
βT1	Low (0.050 mg/L)	6.64%	10.88%
	Medium (1.000 mg/L)	2.34%	4.07%
	High (4.000 mg/L)	2.67%	5.48%
βT2	Low (0.010 mg/L)	3.60%	8.47%
	Medium (1.000 mg/L)	2.65%	5.30%
	High (4.000 mg/L)	2.40%	2.96%
δT2	Low (0.050 mg/L)	10.49%	11.78%
	Medium (0.100 mg/L)	6.86%	6.34%
	High (0.200 mg/L)	1.80%	3.94%
γT10	Low (0.030 mg/L)	3.99%	9.61%
	Medium (1.000 mg/L)	3.10%	5.16%

	High (4.000 mg/L)	4.28%	5.75%
	Low (0.005 mg/L)	4.43%	5.55%
ζT8	Medium (0.050 mg/L)	1.53%	2.92%
	High (0.100 mg/L)	1.68%	2.78%

[a] To assess the reliability of the method, QC samples (standard peptides dissolved by trypsin digested serum) were analyzed. To evaluate inter assay variability of the selected peptides, four concentrations (LLOQ and low, medium, and high QC) were assessed, and each concentration included 5 replicates. For the evaluation of intra assay variability, 6 runs of each level were analyzed on one day, then the tests were performed on 3 consecutive days. Results were displayed as coefficient of variation (CV).

Supplemental **Table 23.** The matrix effects of the DBS samples tested by HPLC-HRMS [a]

Matrix	Tests	Peak area of the selected peptides						
		αT1	αT3	βT1	βT2	γT10	δT2	ζT8
Trypsin digested serum	Sample 1	10062976	879303	6292225	86824241	512418	9711472	17521450
	Sample 2	9877634	903230	6214799	87010023	510056	9455183	17378334
	Sample 3	9374254	979061	6420480	88556858	540430	9576905	17137096
	Sample 4	9556218	895893	6553311	87732019	522684	9944589	17281491
	Sample 5	9312163	907827	6203129	90890517	508121	9523575	17571814
	Sample 6	9982733	888123	6709233	90180854	531341	9726823	17519706
	Mean	9694330	908906	6398863	88532419	520842	9656425	17401649
	CV (%)	3.33	3.95	3.16	1.90	2.50	1.83	0.97
Normal saline	Sample 1	8302722	664161	5669988	93951284	569231	10494054	18912702
	Sample 2	8129314	639732	5953594	99902346	588653	9798125	18853422
	Sample 3	7705823	638648	5844669	98808018	539667	9817648	19280258
	Mean	8045953	647514	5822750	97553883	565850	10036609	19015461
	CV (%)	3.82	2.23	2.46	3.25	4.36	3.95	1.22
Mixed solution (1:1)	Sample 1	8691231	837569	6097312	97778653	511188	10318705	18013566
	Sample 2	9622144	882773	7029342	92732180	557384	10393170	18646478
	Sample 3	10134944	808125	6140682	93353259	562821	10210437	18230553
	Mean	9482773	842822	6422445	94621364	543798	10307437	18296866
	CV (%)	7.72	4.46	8.19	2.91	5.22	0.89	1.76
Total CV (%)		8.93	13.99	6.03	4.99	4.97	3.59	4.08
Matrix effects (%)		6.91	8.30	5.10	1.70	0.08	4.68	0.48

[a] The matrix effects were investigated by comparing the peak area of six native matrix samples prepared using trypsin digested serum, three neat samples prepared using normal saline, and three 1:1 mixtures of native matrix samples with neat samples. The criterion for a passing test is the mean peak area of the 1:1 mixture being within 20% of the average of the measured values of the native and neat matrix solution. Matrix effects= (Average peak area of the native matrix samples and the neat samples - Average peak area of the 1:1 mixture) / Average peak area of the native matrix samples and the neat samples) *100%.

Supplemental **Table 24.** The carryover of the selected peptides detected by HPLC-HRMS [a]

QC samples	Peak area of the selected peptides						
	αT1	αT3	βT1	βT2	γT10	δT2	ζT8
L1	963144	44648	1137439	22411108	136824	2281709	4560085
L2	988244	44640	1243766	22320123	150190	2457500	4722227
L3	1004123	31280	1214422	21594657	111615	2354050	4836930
L4	994842	45083	1121433	21620519	117737	2350083	4952416
L5	835601	39592	1101768	21339375	127755	2408152	4792098
H	15456194	1293999	9352990	180270020	1197188	18911176	39451814
L6	1005680	41485	1133423	21901639	141693	2341261	4644834
L7	899602	40400	1222612	22272344	120192	2287212	4813295
Mean value (L1-L5)	957191	41049	1163766	21857156	128824	2370299	4772751
Carryover rate 1	5.07%	1.06%	2.61%	0.20%	9.99%	1.23%	2.68%
Carryover rate 2	6.02%	1.58%	5.06%	1.90%	6.70%	3.51%	0.85%

[a] The carryover rate of the method was assessed by sequential injection of low and high concentrations of the selected peptides in the sequence of L1-L2-L3-L4-L5-H-L6-L7. Carryover rate 1 = (L6 - Mean value) / mean value*100%. Carryover rate 2 = (L7 - Mean value) / mean value*100%.

Supplemental **Table 25.** The extraction recovery of the selected peptides detected HPLC-HRMS [a]

Selected peptides	Low concentration (+125 μg/L)	Mid concentration （+500 μg/L）	High concentration (+2000 μg/L)
αT1	115.30%	90.63%	96.37%
αT3	79.93%	112.94%	105.42%
βT1	85.07%	100.86%	95.60%
βT2	116.47%	108.01%	104.13%
γT10	92.66%	90.15%	100.74%
**Selected peptides**	**Low concentration (+12.5 μg/L)**	**Mid concentration （+50 μg/L）**	**High concentration (+200 μg/L)**
δT2	84.65%	98.96%	97.89%
ζT8	91.05%	99.15%	98.34%

[a] The extraction recovery of the selected peptides was investigated at 3 concentrations (low, medium, and high). The matrix samples were trypsin digested serum containing standard peptides of 1000.0 μg/L (α_{T1}, α_{T3}, β_{T1}, β_{T2}, and γ_{T10}) or 100.0 μg/L (δ_{T2} and ζ_{T8}). The standard peptides of different concentrations were added into the matrix, then measured for calculating the extraction recovery. The extraction recovery = (Measured concentration of the samples added with standard peptides - Concentration of the matrix samples) / Standard concentration of the added peptides.

Supplemental **Table 26.** Identification of the cases screened positive by HPLC-HRMS and Sebia-CE

Sample number	HPLC–HRMS [a]				Sebia-CE [b]							NGS
	P(α)	P(β)	β_G/β_{T1}	Disease suspected	Hb Bart's (%)	Hb A (%)	Hb F (%)	Hb A2 (%)	Hb A2/Hb A (%)	Abnormal Hb (%)	Disease suspected	Genotypes
0209	1.00	0.00	-	α	3.70	12.50	83.80	0.00	0.00	-	α	$\alpha\alpha/\text{--}^{SEA}$; β^N/β^N
0439	0.99	0.00	-	α	3.20	21.00	75.80	0.00	0.00	-	α	$\alpha\alpha/\text{--}^{SEA}$; β^N/β^N
0572	1.00	0.00	-	α	2.90	21.50	75.60	0.00	0.00	-	α	$\alpha\alpha/\text{--}^{SEA}$; β^N/β^N
0576	0.99	0.00	-	α	2.40	37.10	60.50	0.00	0.00	-	α	$\alpha\alpha/\text{--}^{SEA}$; β^N/β^N
0608	0.98	0.00	-	α	2.00	24.70	73.30	0.00	0.00	-	α	$\alpha\alpha/\text{--}^{SEA}$; β^N/β^N
0968	0.98	0.00	-	α	2.70	22.30	75.00	0.00	0.00	-	α	$\alpha\alpha/\text{--}^{SEA}$; β^N/β^N
1003	0.97	0.00	-	α	1.10	35.40	63.50	0.00	0.00	-	α	$\alpha\alpha/\text{--}^{SEA}$; β^N/β^N
1047	0.95	0.00	-	α	1.90	45.50	52.60	0.00	0.00	-	α	$\alpha\alpha/\text{--}^{SEA}$; β^N/β^N
1060	1.00	0.00	-	α	3.00	15.90	81.10	0.00	0.00	-	α	$\alpha\alpha/\text{--}^{SEA}$; β^N/β^N
1091	0.98	0.00	-	α	0.90	46.20	52.90	0.00	0.00	-	α	$\alpha\alpha/\text{--}^{SEA}$; β^N/β^N
1276	1.00	0.00	-	α	1.70	21.40	76.90	0.00	0.00	-	α	$\alpha\alpha/\text{--}^{SEA}$; β^N/β^N
1463	0.95	0.00	-	α	2.20	34.90	62.90	0.00	0.00	-	α	$\alpha\alpha/\text{--}^{SEA}$; β^N/β^N
1697	1.00	0.00	-	α	2.40	15.80	81.80	0.00	0.00	-	α	$\alpha\alpha/\text{--}^{SEA}$; β^N/β^N
1864	1.00	0.00	-	α	2.50	18.90	78.60	0.00	0.00	-	α	$\alpha\alpha/\text{--}^{SEA}$; β^N/β^N
1921	1.00	0.00	-	α	2.60	16.50	80.90	0.00	0.00	-	α	$\alpha\alpha/\text{--}^{SEA}$; β^N/β^N
2256	1.00	0.00	-	α	2.20	21.50	76.30	0.00	0.00	-	α	$\alpha\alpha/\text{--}^{SEA}$; β^N/β^N
2383	1.00	0.00	-	α	2.20	27.30	70.50	0.00	0.00	-	α	$\alpha\alpha/\text{--}^{SEA}$; β^N/β^N
2399	0.99	0.00	-	α	1.60	19.30	79.10	0.00	0.00	-	α	$\alpha\alpha/\text{--}^{SEA}$; β^N/β^N
2415	1.00	0.00	-	α	1.80	23.10	75.10	0.00	0.00	-	α	$\alpha\alpha/\text{--}^{SEA}$; β^N/β^N
2508	0.99	0.00	-	α	1.00	31.10	67.90	0.00	0.00	-	α	$\alpha\alpha/\text{--}^{SEA}$; β^N/β^N
0427	1.00	0.00	-	α	0.00	16.50	83.50	0.00	0.00	-	normal	$\alpha\alpha/\text{--}^{SEA}$; β^N/β^N

1390	1.00	0.00	-	α	0.00	23.40	76.60	0.00	0.00	-	normal	$\alpha\alpha/--^{SEA}; \beta^N/\beta^N$
1705	1.00	0.00	-	α	0.00	24.40	75.60	0.00	0.00	-	normal	$\alpha\alpha/--^{SEA}; \beta^N/\beta^N$
2590	1.00	0.00	-	α	0.00	21.60	78.40	0.00	0.00	-	normal	$\alpha\alpha/--^{SEA}; \beta^N/\beta^N$
2597	1.00	0.00	-	α	0.00	14.20	85.80	0.00	0.00	-	normal	$\alpha\alpha/--^{SEA}; \beta^N/\beta^N$
2620	0.91	0.08	-	α	0.00	25.20	74.80	0.00	0.00	-	normal	$\alpha\alpha/--^{SEA}; \beta^N/\beta^N$
2633	0.97	0.00	-	α	0.00	21.30	78.70	0.00	0.00	-	normal	$\alpha\alpha/--^{SEA}; \beta^N/\beta^N$
0159	0.95	0.00	-	α	0.00	16.80	83.20	0.00	0.00	-	normal	$\alpha\alpha/-\alpha^{3.7}; \beta^N/\beta^N$
0170	0.46	0.00	-	α	0.00	30.50	69.50	0.00	0.00	-	normal	$\alpha\alpha/-\alpha^{3.7}; \beta^N/\beta^N$
0200	0.37	0.00	-	α	0.20	28.70	71.10	0.00	0.00	-	α	$\alpha\alpha/-\alpha^{3.7}; \beta^N/\beta^N$
0237	0.68	0.01	-	α	0.00	24.30	75.70	0.00	0.00	-	normal	$\alpha\alpha/-\alpha^{3.7}; \beta^N/\beta^N$
1467	0.47	0.00	-	α	0.00	31.40	68.60	0.00	0.00	-	normal	$\alpha\alpha/-\alpha^{3.7}; \beta^N/\beta^N$
1578	0.46	0.00	-	α	0.00	22.30	77.70	0.00	0.00	-	normal	$\alpha\alpha/-\alpha^{3.7}; \beta^N/\beta^N$
1643	0.44	0.00	-	α	0.10	28.20	71.70	0.00	0.00	-	α	$\alpha\alpha/-\alpha^{3.7}; \beta^N/\beta^N$
0058	0.00	0.00	-	normal	0.80	9.70	89.50	0.00	0.00	-	α	$\alpha\alpha/-\alpha^{3.7}; \beta^N/\beta^N$
0128	0.19	0.00	-	normal	0.30	17.90	81.80	0.00	0.00	-	α	$\alpha\alpha/-\alpha^{3.7}; \beta^N/\beta^N$
0389	0.14	0.00	-	normal	0.40	29.90	69.70	0.00	0.00	-	α	$\alpha\alpha/-\alpha^{3.7}; \beta^N/\beta^N$
0547	0.00	0.00	-	normal	0.40	15.00	84.60	0.00	0.00	-	α	$\alpha\alpha/-\alpha^{3.7}; \beta^N/\beta^N$
0582	0.02	0.00	-	normal	0.60	17.00	82.40	0.00	0.00	-	α	$\alpha\alpha/-\alpha^{3.7}; \beta^N/\beta^N$
0720	0.07	0.00	-	normal	0.40	29.20	70.40	0.00	0.00	-	α	$\alpha\alpha/-\alpha^{3.7}; \beta^N/\beta^N$
0877	0.04	0.00	-	normal	0.40	28.40	71.20	0.00	0.00	-	α	$\alpha\alpha/-\alpha^{3.7}; \beta^N/\beta^N$
0890	0.00	0.01	-	normal	0.60	10.70	88.70	0.00	0.00	-	α	$\alpha\alpha/-\alpha^{3.7}; \beta^N/\beta^N$
1009	0.10	0.00	-	normal	0.40	23.20	76.40	0.00	0.00	-	α	$\alpha\alpha/-\alpha^{3.7}; \beta^N/\beta^N$
1159	0.05	0.00	-	normal	0.30	20.90	78.80	0.00	0.00	-	α	$\alpha\alpha/-\alpha^{3.7}; \beta^N/\beta^N$
2432	0.00	0.00	-	normal	0.50	16.90	82.60	0.00	0.00	-	α	$\alpha\alpha/-\alpha^{3.7}; \beta^N/\beta^N$
2694	0.01	0.00	-	normal	0.30	25.50	74.20	0.00	0.00	-	α	$\alpha\alpha/-\alpha^{3.7}; \beta^N/\beta^N$
1819	0.95	1.00	-	α and β	0.00	70.50	28.20	1.30	0.02	-	normal	$\alpha\alpha/-\alpha^{4.2}; \beta^N/\beta^N$

1820	1.00	1.00	-	α and β	0.10	26.20	73.70	0.00	0.00	-	α	$\alpha\alpha/-\alpha^{4.2}; \beta^{N}/\beta^{N}$
1622	0.10	0.00	-	normal	0.20	18.90	80.90	0.00	0.00	-	α	$\alpha\alpha/-\alpha^{4.2}; \beta^{N}/\beta^{N}$
1817	0.46	0.00	-	α	0.00	56.00	43.20	0.80	0.01	-	normal	$\alpha\alpha/\alpha^{WS}\alpha; \beta^{N}/\beta^{N}$
0218	0.57	0.01	-	α	0.00	46.00	53.10	0.90	0.02	-	normal	$\alpha\alpha/\alpha^{CS}\alpha; \beta^{N}/\beta^{N}$
0265	0.00	0.00	-	normal	1.30	8.30	90.40	0.00	0.00	-	α	$\alpha\alpha/\alpha^{CS}\alpha; \beta^{N}/\beta^{N}$
0641	0.00	0.00	-	normal	1.50	11.70	86.80	0.00	0.00	-	α	$\alpha\alpha/\alpha^{CS}\alpha; \beta^{N}/\beta^{N}$
2092	0.01	0.00	-	normal	0.50	20.90	78.60	0.00	0.00	-	α	$\alpha\alpha/\alpha^{CS}\alpha; \beta^{N}/\beta^{N}$
2331	0.00	0.01	-	normal	0.80	12.60	86.60	0.00	0.00	-	α	$\alpha\alpha/\alpha^{CS}\alpha; \beta^{N}/\beta^{N}$
1639	0.15	0.00	-	normal	2.10	18.30	79.60	0.00	0.00	-	α	$-\alpha^{3.7}/-\alpha^{3.7}; \beta^{N}/\beta^{N}$
1747	0.08	0.00	-	normal	2.10	20.40	77.50	0.00	0.00	-	α	$-\alpha^{3.7}/-\alpha^{3.7}; \beta^{N}/\beta^{N}$
0030	0.62	0.00	-	α	0.00	15.70	84.30	0.00	0.00	-	normal	$\alpha\alpha\alpha^{anti3.7}/\alpha\alpha; \beta^{N}/\beta^{N}$
2586	0.00	0.50	-	β	0.00	27.70	72.30	0.00	0.00	-	normal	$\alpha\alpha\alpha^{anti3.7}/\alpha\alpha; \beta^{N}/\beta^{N}$
0811	1.00	0.00	-	α	0.00	7.40	92.30	0.30	0.04	-	β	$\alpha\alpha\alpha^{anti4.2}/\alpha\alpha; \beta^{N}/\beta^{N}$
1816	0.03	1.00	-	β	0.00	36.30	63.10	0.60	0.02	-	normal	$\alpha\alpha\alpha^{anti4.2}/\alpha\alpha; \beta^{N}/\beta^{N}$
1818	0.29	1.00	-	β	0.00	10.30	89.70	0.00	0.00	-	normal	$\alpha\alpha\alpha^{anti4.2}/\alpha\alpha; \beta^{N}/\beta^{N}$
0351	0.00	0.03	-	normal	0.00	9.30	90.60	0.10	0.01	-	β	$\alpha\alpha\alpha^{anti4.2}/\alpha\alpha; \beta^{N}/\beta^{N}$
1740	0.92	0.00	-	α	0.00	31.20	68.80	0.00	0.00	-	normal	$\alpha\alpha\alpha^{anti3.7}/\text{--SEA}; \beta^{N}/\beta^{N}$
0514	0.00	0.41	-	β	0.00	10.80	89.00	0.20	0.02	-	β	$\alpha\alpha/\alpha\alpha; \beta^{N}/\beta^{Codon\ 17}$
0654	0.00	0.61	-	β	0.00	6.70	93.30	0.00	0.00	-	β	$\alpha\alpha/\alpha\alpha; \beta^{N}/\beta^{Codon\ 17}$
1240	0.00	0.92	-	β	0.00	6.70	93.30	0.00	0.00	-	β	$\alpha\alpha/\alpha\alpha; \beta^{N}/\beta^{Codon\ 17}$
1241	0.00	0.98	-	β	0.00	6.80	93.20	0.00	0.00	-	β	$\alpha\alpha/\alpha\alpha; \beta^{N}/\beta^{Codon\ 17}$
1515	0.00	0.98	-	β	0.00	5.50	94.50	0.00	0.00	-	β	$\alpha\alpha/\alpha\alpha; \beta^{N}/\beta^{Codon\ 17}$
0063	0.04	0.99	-	β	0.00	21.30	78.70	0.00	0.00	-	normal	$\alpha\alpha/\alpha\alpha; \beta^{N}/\beta^{Codon\ 17}$
0189	0.07	0.94	-	β	0.00	13.30	86.70	0.00	0.00	-	normal	$\alpha\alpha/\alpha\alpha; \beta^{N}/\beta^{Codon\ 17}$
0536	0.00	0.93	-	β	0.00	10.30	89.70	0.00	0.00	-	normal	$\alpha\alpha/\alpha\alpha; \beta^{N}/\beta^{Codon\ 17}$
0642	0.00	0.39	-	β	0.00	10.10	89.90	0.00	0.00	-	normal	$\alpha\alpha/\alpha\alpha; \beta^{N}/\beta^{Codon\ 17}$

0909	0.00	0.80	-	β	0.00	11.90	88.10	0.00	0.00	-	normal	αα/αα; $\beta^{N}/\beta^{Codon\ 17}$
1012	0.00	0.36	-	β	0.00	10.90	89.10	0.00	0.00	-	normal	αα/αα; $\beta^{N}/\beta^{Codon\ 17}$
1847	0.02	0.95	-	β	0.00	13.90	86.10	0.00	0.00	-	normal	αα/αα; $\beta^{N}/\beta^{Codon\ 17}$
2445	0.02	0.95	-	β	0.00	15.40	84.60	0.00	0.00	-	normal	αα/αα; $\beta^{N}/\beta^{Codon\ 17}$
0396	0.00	0.16	-	β	0.00	9.00	90.90	0.10	0.01	-	β	αα/αα; $\beta^{N}/\beta^{IVS-II-654}$
0670	0.00	0.88	-	β	0.00	6.30	93.70	0.00	0.00	-	β	αα/αα; $\beta^{N}/\beta^{IVS-II-654}$
0707	0.00	0.96	-	β	0.00	8.70	91.10	0.20	0.02	-	β	αα/αα; $\beta^{N}/\beta^{IVS-II-654}$
0814	0.00	1.00	-	β	0.00	5.30	94.70	0.00	0.00	-	β	αα/αα; $\beta^{N}/\beta^{IVS-II-654}$
1634	0.00	0.99	-	β	0.00	4.40	95.60	0.00	0.00	-	β	αα/αα; $\beta^{N}/\beta^{IVS-II-654}$
0284	0.00	0.28	-	β	0.00	12.20	87.80	0.00	0.00	-	normal	αα/αα; $\beta^{N}/\beta^{IVS-II-654}$
0821	0.00	0.63	-	β	0.00	12.70	87.30	0.00	0.00	-	normal	αα/αα; $\beta^{N}/\beta^{IVS-II-654}$
1043	0.00	0.39	-	β	0.00	13.70	86.30	0.00	0.00	-	normal	αα/αα; $\beta^{N}/\beta^{IVS-II-654}$
1314	0.00	0.45	-	β	0.00	15.90	84.10	0.00	0.00	-	normal	αα/αα; $\beta^{N}/\beta^{IVS-II-654}$
1329	0.00	0.26	-	β	0.00	12.50	87.50	0.00	0.00	-	normal	αα/αα; $\beta^{N}/\beta^{IVS-II-654}$
1355	0.00	0.28	-	β	0.00	10.90	89.10	0.00	0.00	-	normal	αα/αα; $\beta^{N}/\beta^{IVS-II-654}$
1409	0.00	0.60	-	β	0.00	12.60	87.40	0.00	0.00	-	normal	αα/αα; $\beta^{N}/\beta^{IVS-II-654}$
1836	0.00	0.77	-	β	0.00	8.70	91.30	0.00	0.00	-	normal	αα/αα; $\beta^{N}/\beta^{IVS-II-654}$
0404	0.00	0.90	-	β	0.00	8.50	91.40	0.10	0.01	-	β	αα/αα; $\beta^{N}/\beta^{Codons\ 41/42}$
0556	0.00	0.99	-	β	0.00	9.30	90.60	0.10	0.01	-	β	αα/αα; $\beta^{N}/\beta^{Codons\ 41/42}$
0651	0.00	0.38	-	β	0.00	8.40	91.50	0.10	0.01	-	β	αα/αα; $\beta^{N}/\beta^{Codons\ 41/42}$
0660	0.00	0.90	-	β	0.00	8.60	91.30	0.10	0.01	-	β	αα/αα; $\beta^{N}/\beta^{Codons\ 41/42}$
0750	0.00	0.32	-	β	0.00	7.00	93.00	0.00	0.00	-	β	αα/αα; $\beta^{N}/\beta^{Codons\ 41/42}$
0964	0.00	0.09	-	normal	0.00	8.90	91.00	0.10	0.01	-	β	αα/αα; $\beta^{N}/\beta^{Codons\ 41/42}$
1512	0.00	0.23	-	β	0.00	11.20	88.80	0.00	0.00	-	normal	αα/αα; $\beta^{N}/\beta^{Codons\ 41/42}$
1861	0.00	0.66	-	β	0.00	11.30	88.70	0.00	0.00	-	normal	αα/αα; $\beta^{N}/\beta^{Codons\ 41/42}$
2048	0.00	0.86	-	β	0.00	8.10	91.90	0.00	0.00	-	normal	αα/αα; $\beta^{N}/\beta^{Codons\ 41/42}$

2110	0.00	0.96	-	β	0.00	10.20	89.80	0.00	0.00	-	normal	αα/αα; $\beta^{N}/\beta^{Codons\ 41/42}$
2474	0.00	0.76	-	β	0.00	12.40	87.60	0.00	0.00	-	normal	αα/αα; $\beta^{N}/\beta^{Codons\ 41/42}$
1300	0.00	0.28	-	β	0.00	8.60	91.40	0.00	0.00	-	normal	αα/αα; $\beta^{N}/\beta^{Codons\ 27/28}$
2009	0.00	0.16	-	β	0.00	11.40	88.60	0.00	0.00	-	normal	αα/αα; $\beta^{N}/\beta^{Codons\ 71/72}$
2086	0.00	0.25	-	β	0.00	13.00	87.00	0.00	0.00	-	normal	αα/αα; Chinese $^{G}\gamma^{+}(^{A}\gamma\delta\beta)$
0325	0.02	0.87	-	β	0.00	8.50	91.50	0.00	0.00	-	normal	$\alpha\alpha\alpha^{anti3.7}/\alpha\alpha$; $\beta^{N}/\beta^{IVS\text{-}II\text{-}654}$
0863	0.00	0.64	-	β	0.00	14.00	86.00	0.00	0.00	-	normal	HKαα/αα; $\beta^{N}/\beta^{Codon\ 17}$
0756	0.00	0.00	0.18	Hb D-Los Angeles	0.00	7.00	90.30	0.00	0.00	2.70	Hb D/G/K	Hb D-Los Angeles
0772	0.00	0.00	0.21	Hb D-Los Angeles	0.00	7.30	90.30	0.00	0.00	2.40	Hb D/G/K	Hb D-Los Angeles

[a] HPLC－HRMS decision rules: P(α) ≥ 0.33 indicates α-thalassemia; P(β) ≥ 0.33 indicates β-thalassemia; β_{G}/β_{T1} > 0.00 indicates abnormal Hb disorders.

[b] See Supplementary Table 4 for Sebia CE decision rules.
